# Supplementary material for: A transcriptional response to replication stress selectively expands a subset of Brca2-mutant mammary epithelial cells
Source: Nat Commun. 2023 Aug 25;14:5206. doi: 10.1038/s41467-023-40956-w (PMC10457340; doi:10.1038/s41467-023-40956-w)
Supplement: Supplementary file 1 — Supplementary Information [file 41467_2023_40956_MOESM1_ESM.pdf]

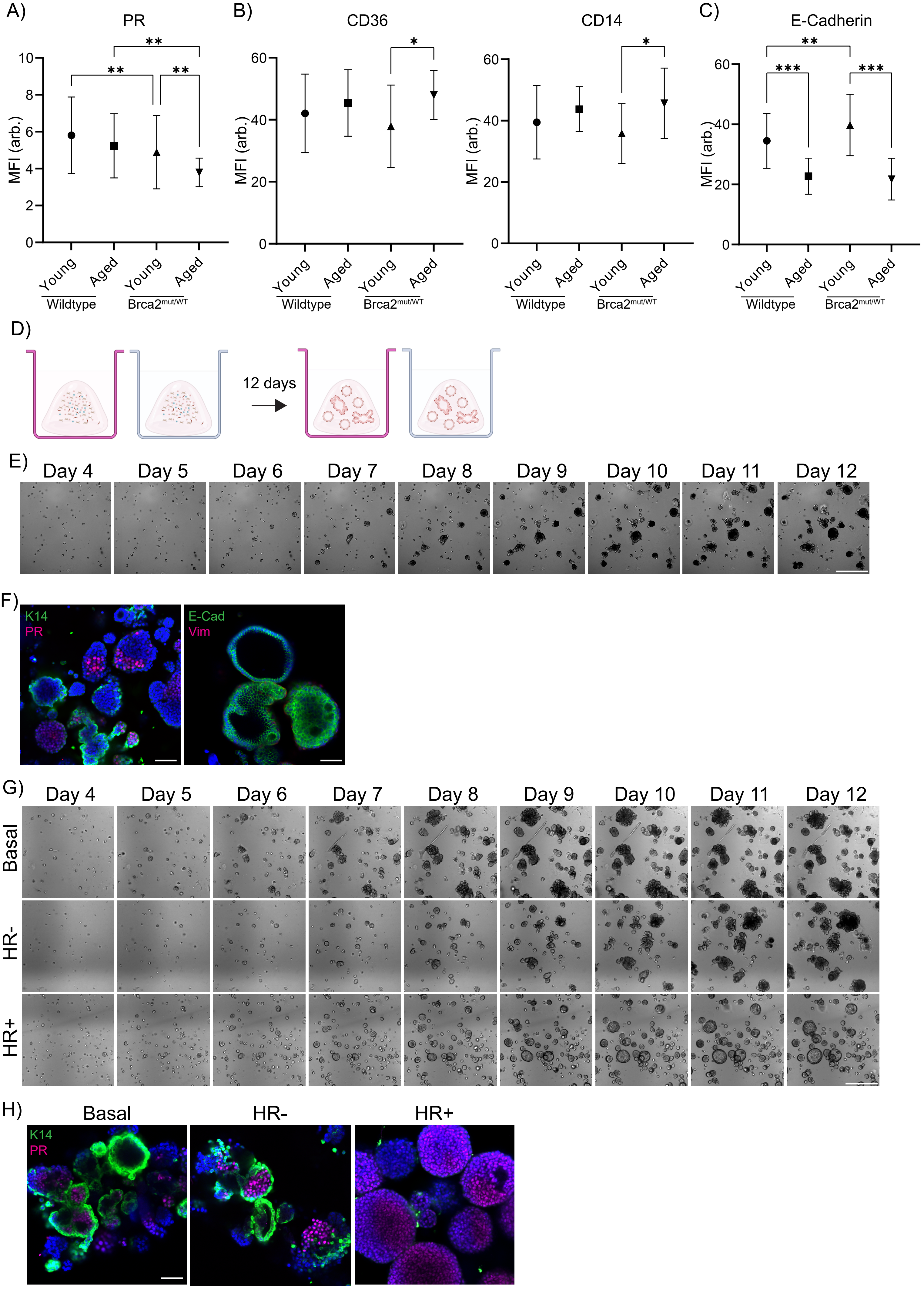

A)
PR
MFI (arb.)
Young
Aged
Young
Aged
Wildtype
Brca2mut/WT
B)
CD36
CD14
MFI (arb.)
C)
E-Cadherin
MFI (arb.)
D)
12 days
E)
Day 4
Day 5
Day 6
Day 7
Day 8
Day 9
Day 10
Day 11
Day 12
F)
K14
PR
E-Cad
Vim
G)
Basal
HR-
HR+
H)
Basal
HR-
HR+
K14
PR

Supplementary Figure 1. **Characteristics of mammary epithelial organoids**. Signal intensities of A)PR B) CD36 and CD14, and C) E-Cadherin. All epithelial ducts (60-210) from a whole mammary gland were imaged for each antibody and mean fluorescent intensities (MFI) values reported in arbitrary units (arb). Data presented mean +/- SD (n=3). Kruskal-Wallis followed by Dunn's multiple comparison test was performed. A) **p=0.004, Source data provided. B) CD36 *p= 0.023, CD14 *p=0.0109, Source data provided. C) E-Cadherin ***p=0.0005, **p=0.005. Source data provided. D) Schematic illustrating the experimental pipeline of culturing mammary epithelial organoids. Created with BioRender.com. E) Brightfield timeline images of mammary cells detailing propagation of organoid development after seeding as non-sorted single cells. Representative images from three independent experiments. Scale bar = 800µm. F) Representative immunofluorescent images of mammary gland organoids after 12 days culturing. K14 (green), PR (magenta). Representative images on the left and E-Cadherin (green) and Vimentin (magenta) representative images on the right. Nuclear staining with DAPI (blue). Representative images from three independent experiments. Scale bars, 50 µm. G) Brightfield images of Basal, HR- luminal and HR+ luminal flow sorted cells after seeding (P0) and imaged daily. Representative images from three independent experiments. Scale bar = 800µm. H) Representative immunofluorescent images of mammary gland organoids derived from Basal (left), HR- luminal (middle) or HR+ luminal cells after 12 days culturing. K14 (green), PR (magenta), nuclear staining with DAPI (blue). Representative images from three independent experiments. Scale bars, 50 µm.

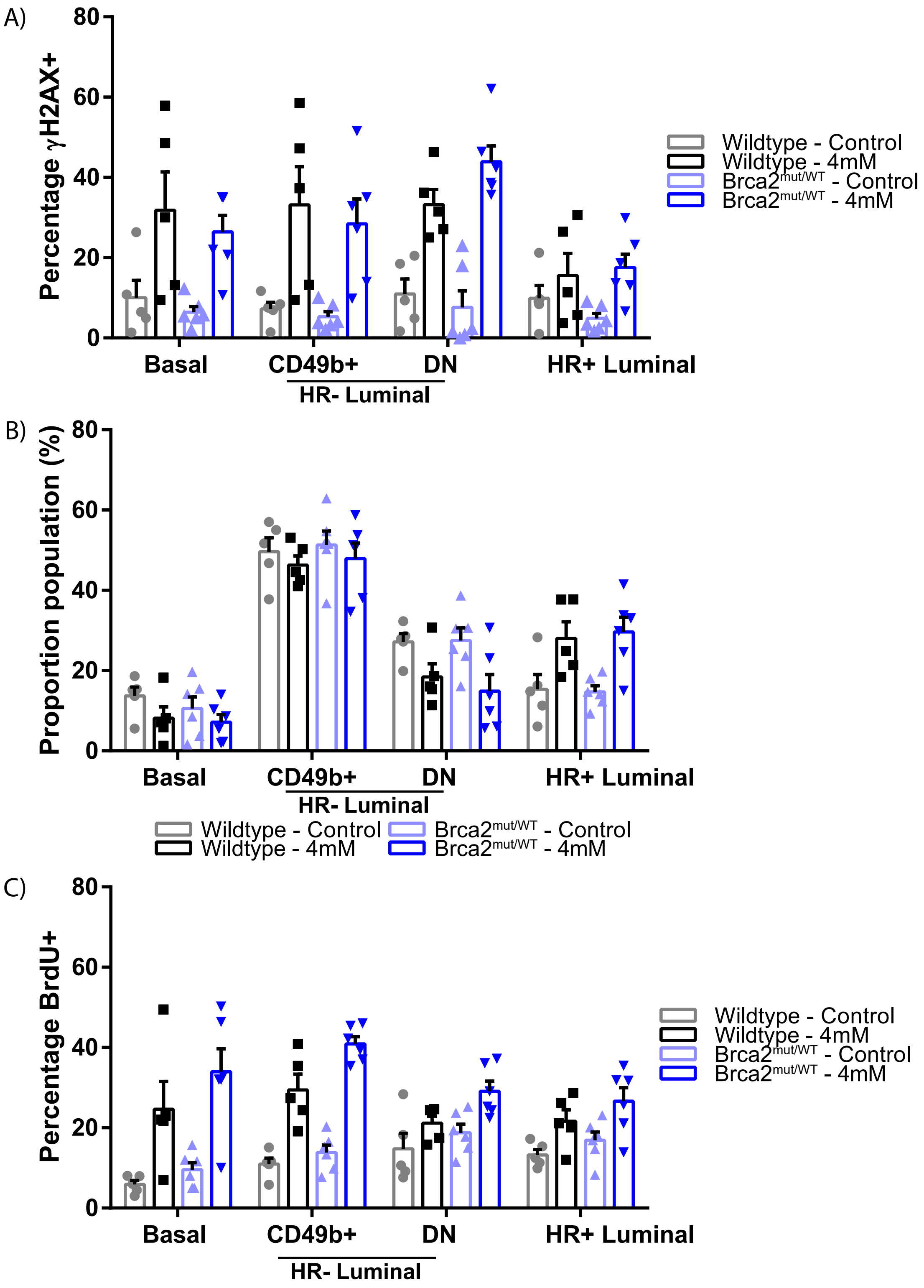
A)
Percentage γH2AX+
0
20
40
60
80
Basal
CD49b+
DN
HR+ Luminal
HR- Luminal
Wildtype - Control
Wildtype - 4mM
Brca2mut/WT - Control
Brca2mut/WT - 4mM
B)
Proportion population (%)
0
20
40
60
80
Basal
CD49b+
DN
HR+ Luminal
HR- Luminal
Wildtype - Control
Wildtype - 4mM
Brca2mut/WT - Control
Brca2mut/WT - 4mM
C)
Percentage BrdU+
0
20
40
60
80
Basal
CD49b+
DN
HR+ Luminal
HR- Luminal
Wildtype - Control
Wildtype - 4mM
Brca2mut/WT - Control
Brca2mut/WT - 4mM

Supplementary Figure 2. **Brca2$^{mut/WT}$ mammary organoids have similar response to short term DNA genotoxic stress at high concentrations**. A) Quantification of the percentage of γH2AX+ cells from wildtype or Brca2$^{mut/WT}$ organoids after 4mM HU treatment. Data depicts the different epithelial populations. Data presented as the mean +/- SEM (n=5-6). Source data provided. B) Quantification of the proportion of epithelial populations in the wildtype or Brca2$^{mut/WT}$ organoids after 4mM HU treatment. Data presented as mean +/- SEM (n=5-6). Source data provided. C) Quantification of the percentage of BrdU+ cells in the different epithelial subpopulations from wildtype or Brca2$^{mut/WT}$ organoids after 4mM HU treatment. Data presented as mean +/- SEM (n=5-6). An ANOVA followed by Fishers LSD showed no significant differences. Source data are provided as a Source data file.

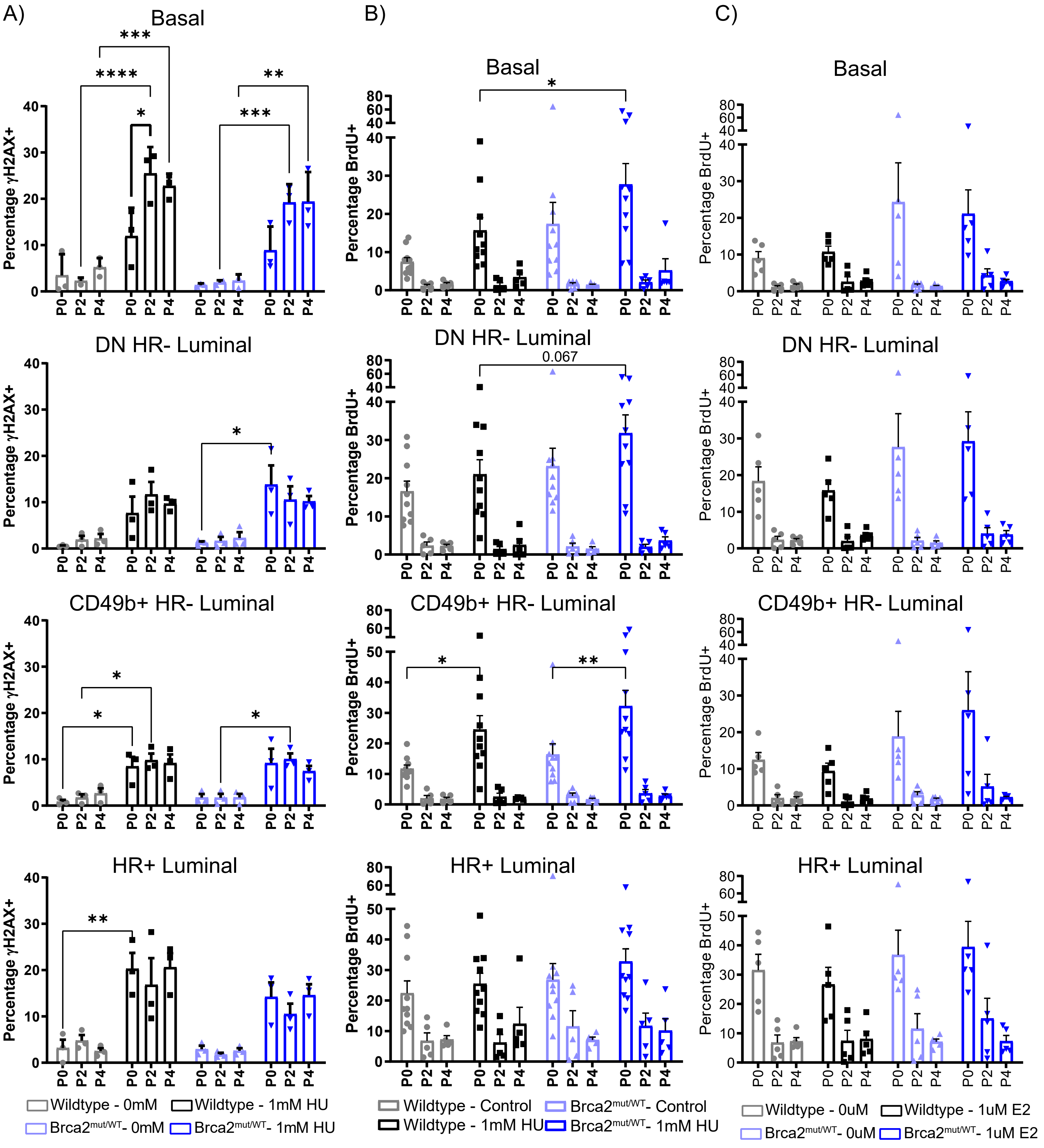
A)
B)
C)
Basal
DN HR- Luminal
CD49b+ HR- Luminal
HR+ Luminal
Percentage γH2AX+
Percentage BrdU+
0.067
P0
P2
P4
Wildtype - 0mM
Wildtype - 1mM HU
Brca2mut/WT- 0mM
Brca2mut/WT- 1mM HU
Wildtype - Control
Wildtype - 1mM HU
Brca2mut/WT- Control
Brca2mut/WT- 1mM HU
Wildtype - 0uM
Wildtype - 1uM E2
Brca2mut/WT- 0uM
Brca2mut/WT- 1uM E2

Supplementary Figure 3. **Brca2$^{mut/WT}$ or wildtype mammary organoids response to DNA genotoxic stresses over time.** A) Quantification of the percentage of γH2AX+ cells from wildtype or Brca2$^{mut/WT}$ organoids after 1mM HU treatment over multiple passages (P0, P2 and P4). Data depicts the different epithelial populations. Data presented as the mean +/- SEM (n=3). An ANOVA followed by a Fishers LSD test was performed. Source data provided. B) Quantification of the percentage of BrdU+ cells from wildtype or Brca2$^{mut/WT}$ organoids after 1mM HU treatment over multiple passages (P0, P2 and P4). Data depicts the different epithelial populations. Data presented as the mean +/- SEM (n=9-10). An ANOVA followed by a Fishers LSD test was performed. Source data provided. C) Quantification of the percentage of BrdU+ cells from wildtype or Brca2$^{mut/WT}$ organoids after 1µM E2 treatment over multiple passages (P0, P2 and P4). Data depicts the different epithelial populations. Data presented as the mean +/- SEM (n=5). An ANOVA followed by a Fishers LSD test showed no statistical significance. Source data are provided as a Source data file.

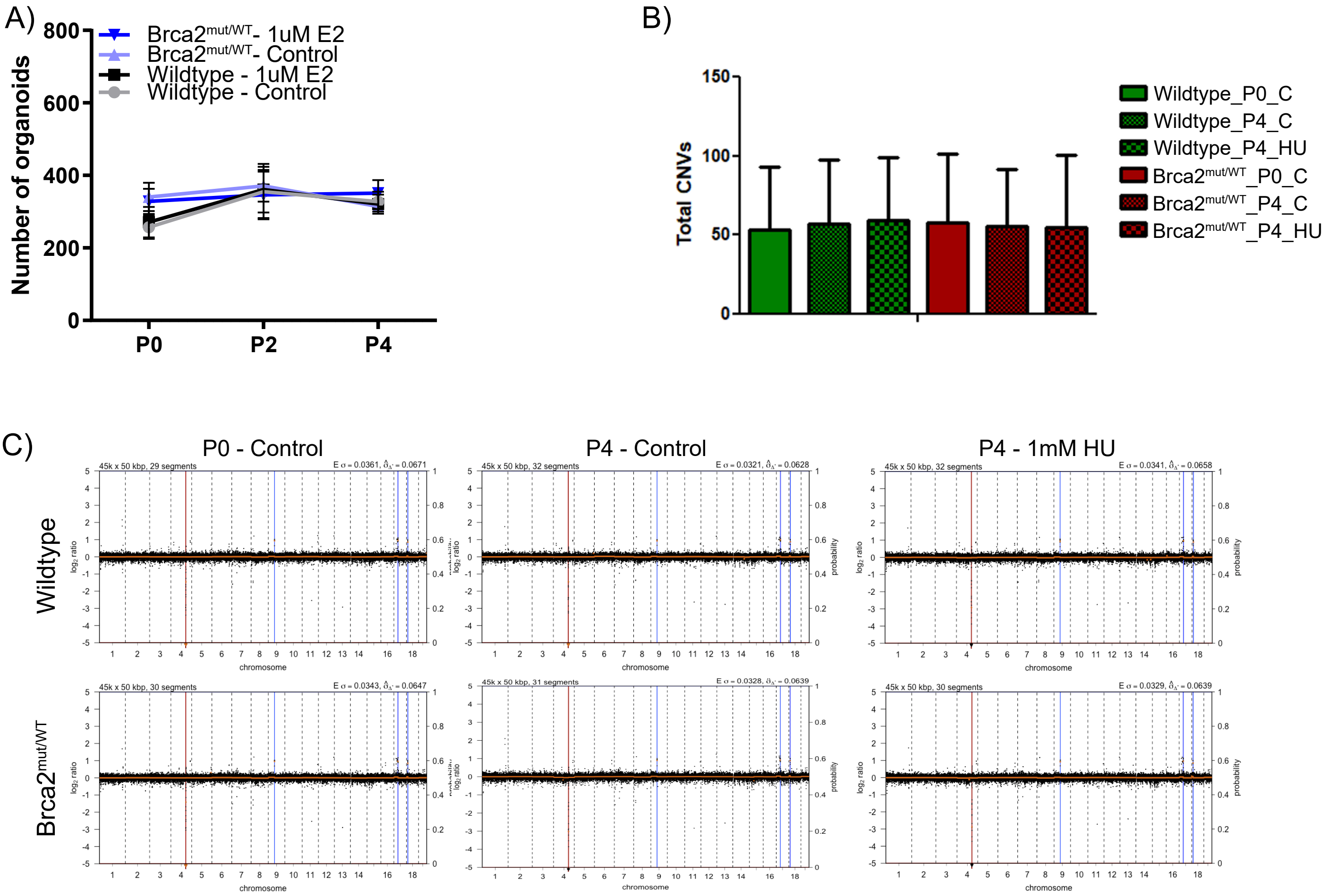
A)
Number of organoids
800
600
400
200
0
P0
P2
P4
Brca2mut/WT- 1uM E2
Brca2mut/WT- Control
Wildtype - 1uM E2
Wildtype - Control
B)
Total CNVs
150
100
50
0
Wildtype_P0_C
Wildtype_P4_C
Wildtype_P4_HU
Brca2mut/WT_P0_C
Brca2mut/WT_P4_C
Brca2mut/WT_P4_HU
C)
P0 - Control
P4 - Control
P4 - 1mM HU
Wildtype
Brca2mut/WT
45k x 50 kbp, 29 segments
45k x 50 kbp, 32 segments
45k x 50 kbp, 30 segments
45k x 50 kbp, 31 segments
chromosome
probability

Supplementary Figure 4. **CNV analysis of passage 4 HU treated organoids.** A) Quantification of the number of wildtype or Brca2$^{mut/WT}$ organoids formed over multiple passages (P0, P2 and P4) following E2 treatments. Data presented as mean +/- SEM (n=5 per group). Mann-Whitney 2 tailed unpaired test showed no statistical significance. Source data provided. B) Bar chart of the total CNVs detected of sWGS in wildtype or Brca2$^{mut/WT}$ organoids from passage 0 and passage 4. Data presented as mean +/- SEM (n=3). Source data provided. C) aCGH plots from wildtype (upper) or Brca2$^{mut/WT}$ (lower) organoids at passage 0 and passage 4. Somatic CNAs were absent in all organoids. Representative plots from 3 independent experiments are shown.

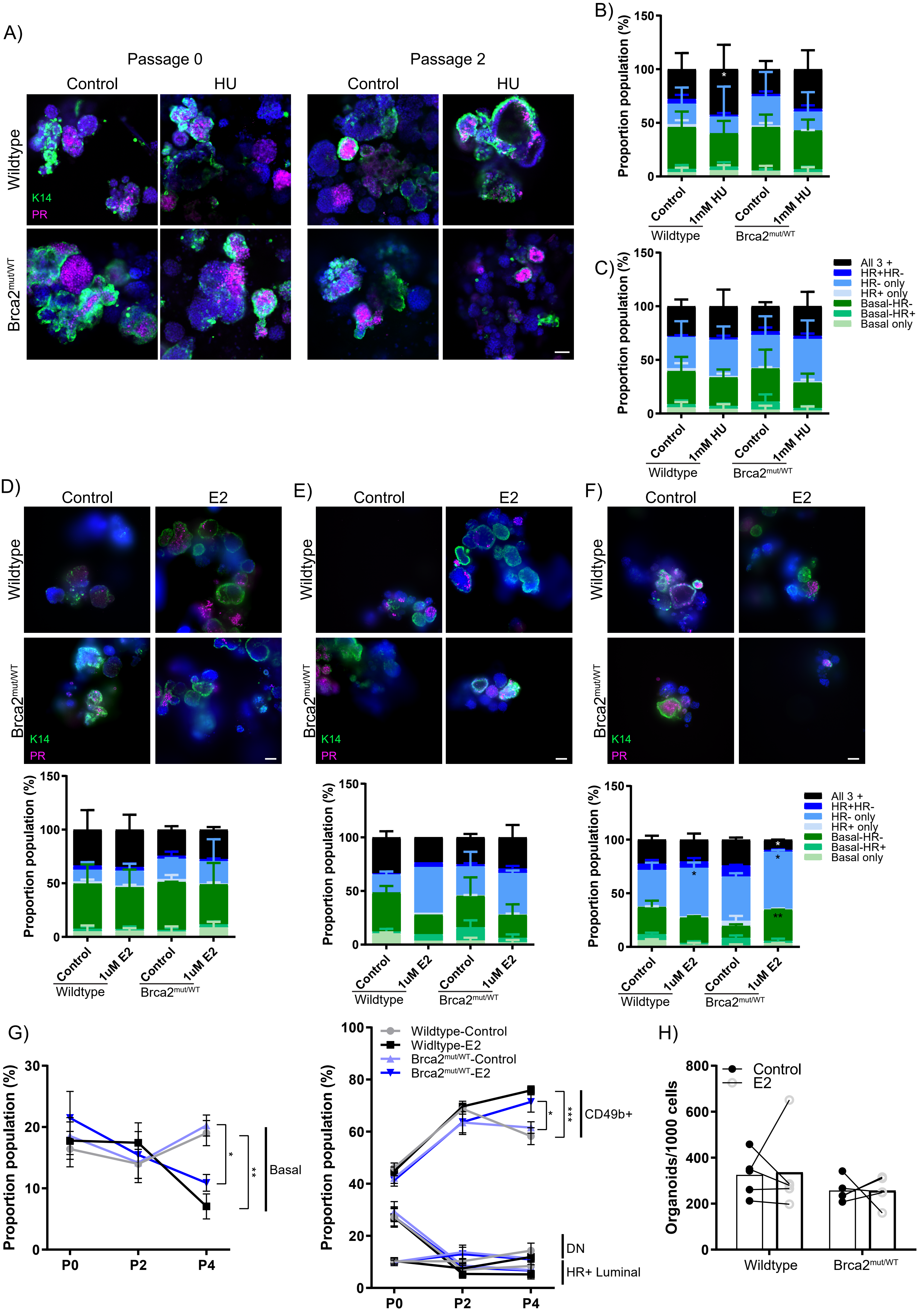

A)
Passage 0
Passage 2
Control
HU
Wildtype
Brca2mut/WT
K14
PR
B)
C)
Proportion population (%)
Control
1mM HU
All 3 +
HR+HR-
HR- only
HR+ only
Basal-HR-
Basal-HR+
Basal only
D)
E)
F)
E2
1uM E2
G)
P0
P2
P4
Basal
Wildtype-Control
Widltype-E2
Brca2mut/WT-Control
Brca2mut/WT-E2
CD49b+
DN
HR+ Luminal
H)
Organoids/1000 cells

Supplementary Figure 5. **Analysis of wildtype or Brca2$^{mut/WT}$ organoids treated with genotoxic stress over time.** A) Representative immunofluorescent images (left) of wildtype or Brca2$^{mut/WT}$ organoids at Passage 0 and passage 2 after control or HU treatments. K14 (green), PR (magenta) and DAPI (blue). Scale bars, 50 µm. (B-C) Quantification of immunofluorescent images of wildtype or Brca2$^{mut/WT}$ organoids post HU treatment at B) passage 0 or C) passage 2. Data presented as mean +/- SD (n=3). An ANOVA followed by Tukey's multiple comparison test showed no statistical differences. Source data provided. (D-F) Quantification of immunofluorescent images of wildtype or Brca2$^{mut/WT}$ organoids post E2 treatment stained with K14 (green), PR (magenta) and DAPI (blue) at D) passage 0, E) passage 2 or F) passage 4. Data presented as mean +/- SD (n=3). An ANOVA followed by Tukey's multiple comparison test was performed. Passage 4 wildtype-E2 HR- only * p= 0.0149; Brca2$^{mut/WT}$-E2 All 3+ * p= 0.0117, HR- only * p= 0.0350 and Basal+HR- ** p= 0.0021.
G) Quantification of the percentage of basal (left) and luminal (right) cell types formed over multiple passages (P0, P2, P4) from wildtype or Brca2$^{mut/WT}$ organoids treated with E2. Data presented as mean +/- SEM n=5. Mann-Whitney two-tailed unpaired t-test was used. Basal * p =0.0261, ** p = 0. 0.0051; Luminal *p = 0.0169, *** p < 0.001. Source data provided. H) Quantification of the number of organoids formed from wildtype or Brca2$^{mut/WT}$ single cells after 5 intermittent E2 treatments. Data presented as mean +/- SEM (n=5). Student's t-test was performed and no statistical significance observed. Source data are provided as a Source data file.

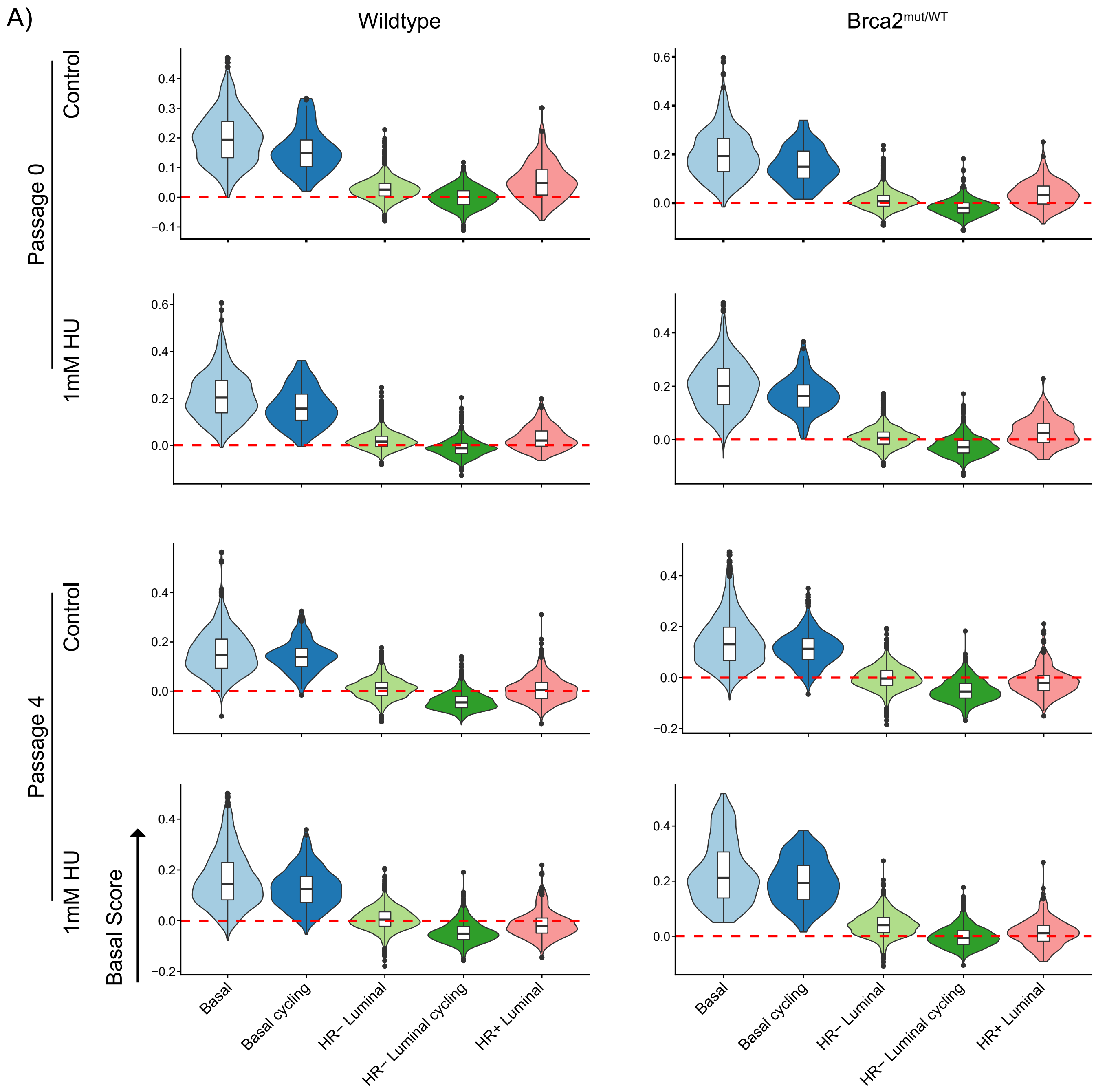

A)
Wildtype
Brca2mut/WT
Passage 0
Control
1mM HU
Passage 4
Control
1mM HU
Basal Score
Basal
Basal cycling
HR− Luminal
HR− Luminal cycling
HR+ Luminal

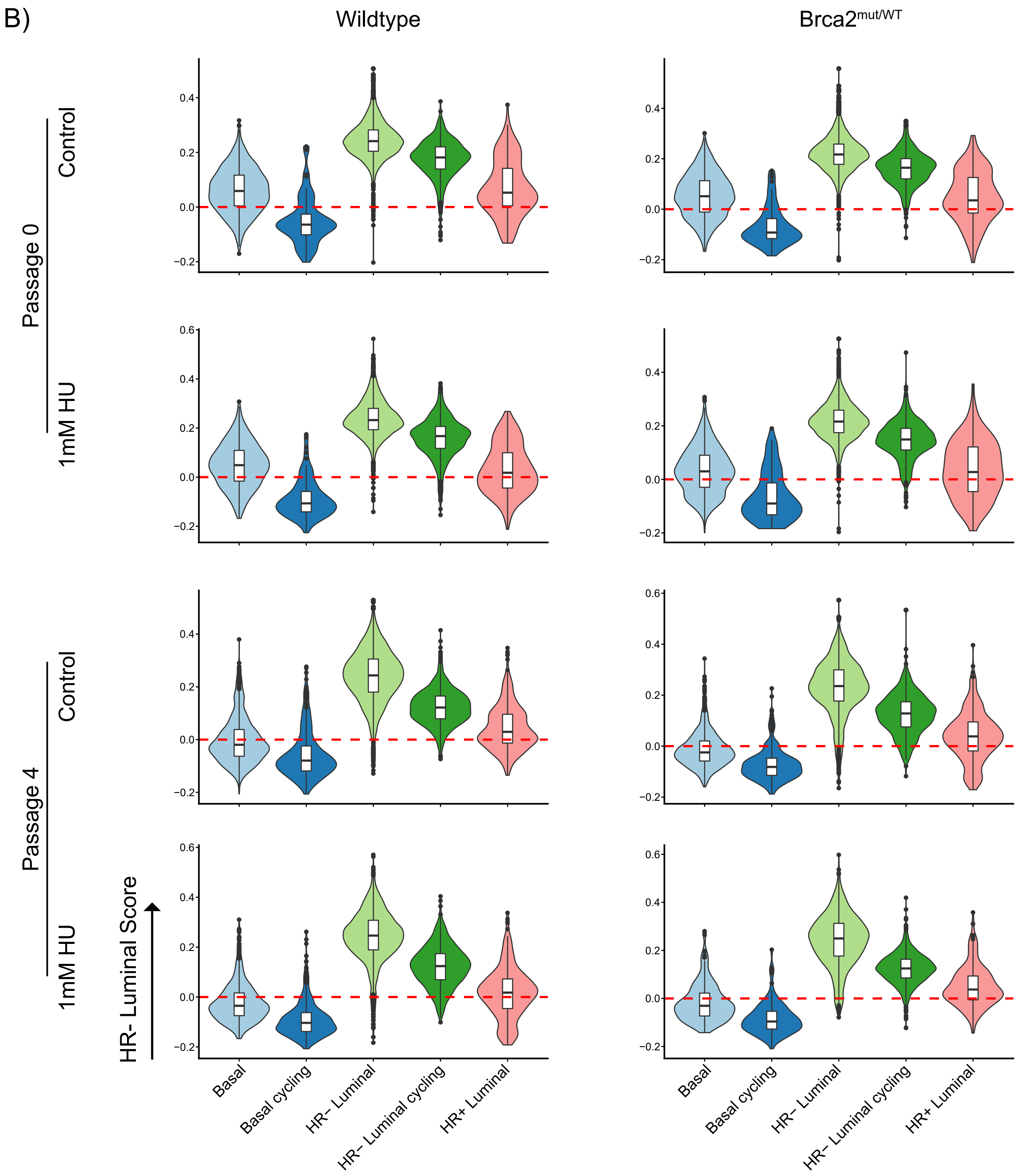

B)
Wildtype
Brca2mut/WT
Passage 0
Control
1mM HU
Passage 4
Control
1mM HU
HR- Luminal Score
0.6
0.4
0.2
0.0
−0.2
Basal
Basal cycling
HR− Luminal
HR− Luminal cycling
HR+ Luminal

C)

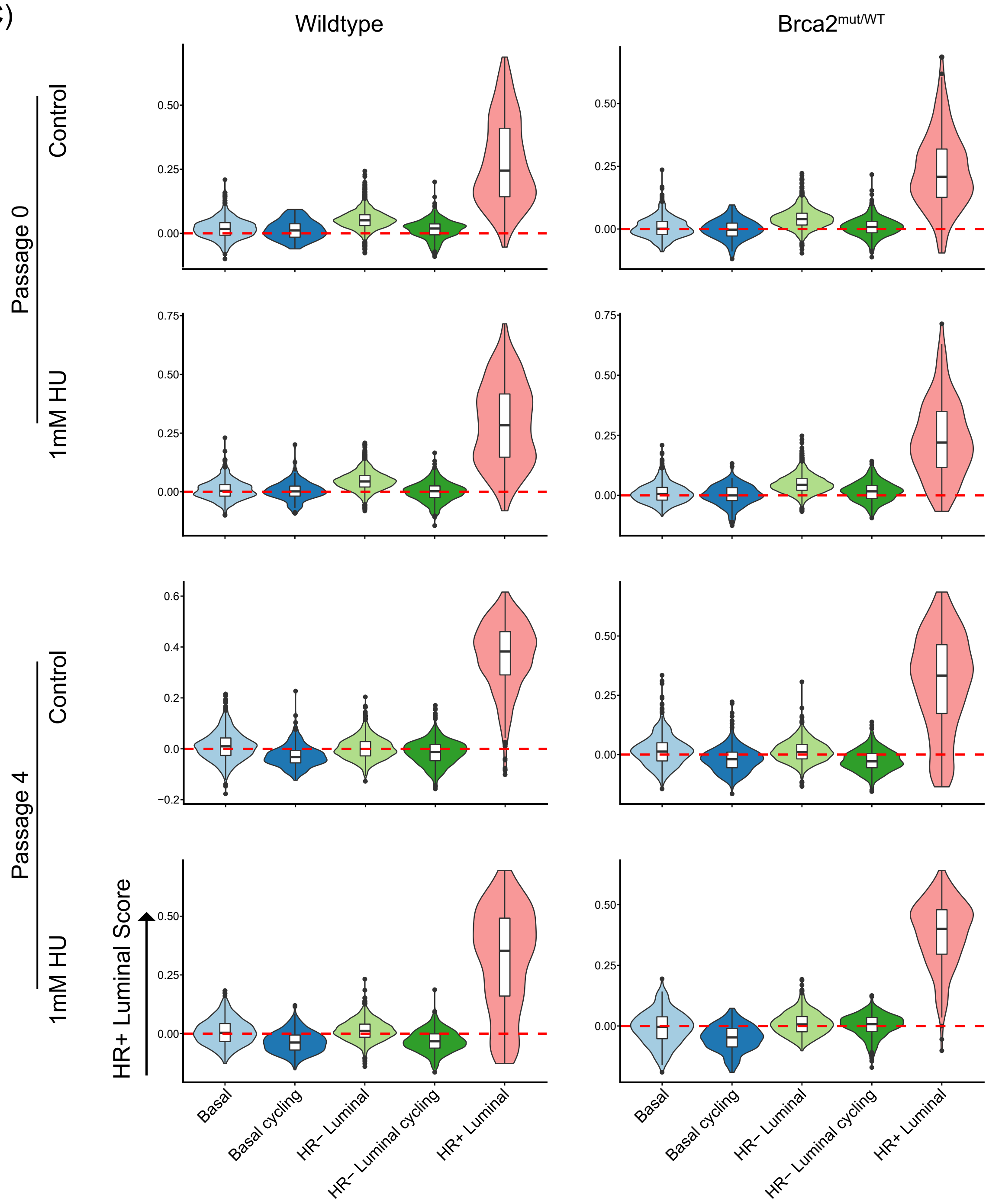

Wildtype
Brca2mut/WT
Passage 0
Control
1mM HU
Passage 4
Control
1mM HU
HR+ Luminal Score
0.75
0.50
0.25
0.00
0.6
0.4
0.2
0.0
−0.2
Basal
Basal cycling
HR− Luminal
HR− Luminal cycling
HR+ Luminal

Supplementary Figure 6. **Mammary epithelial signature scores in cells from organoid clusters**. Violin plots of A) Basal, B) HR- Luminal and C) HR+ Luminal cell scores across the mammary cell clusters of wildtype or Brca2$^{mut/WT}$ unchallenged and HU treated organoids from passage 0 or 4.

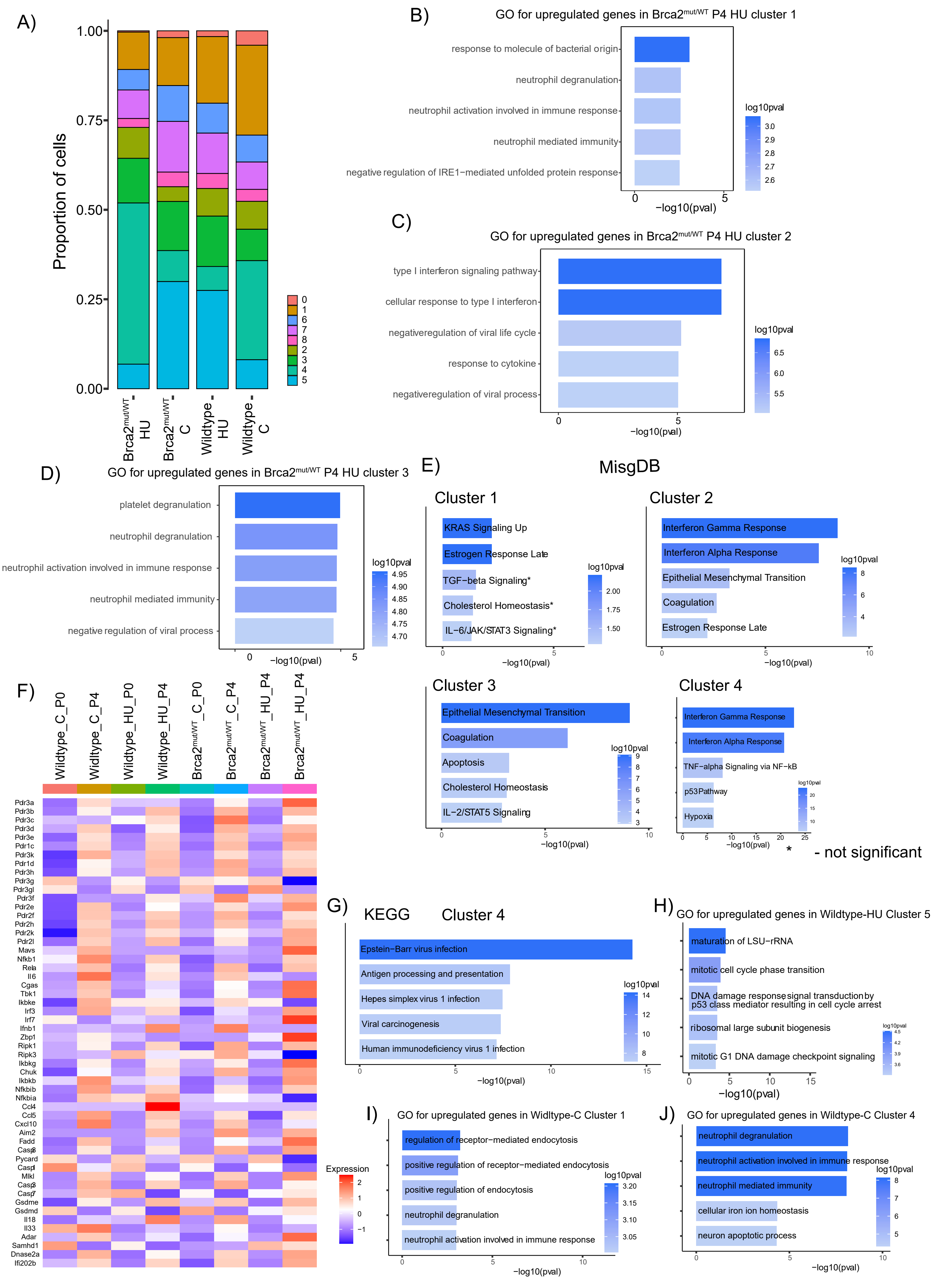
A)
Proportion of cells
1.00
0.75
0.50
0.25
0.00
0
1
6
7
8
2
3
4
5
Brca2mut/WT HU
Brca2mut/WT C
Wildtype HU
Wildtype C
B)
GO for upregulated genes in Brca2mut/WT P4 HU cluster 1
response to molecule of bacterial origin
neutrophil degranulation
neutrophil activation involved in immune response
neutrophil mediated immunity
negative regulation of IRE1−mediated unfolded protein response
log10pval
3.0
2.9
2.8
2.7
2.6
0
5
−log10(pval)
C)
GO for upregulated genes in Brca2mut/WT P4 HU cluster 2
type I interferon signaling pathway
cellular response to type I interferon
negativeregulation of viral life cycle
response to cytokine
negativeregulation of viral process
log10pval
6.5
6.0
5.5
0
5
−log10(pval)
D)
GO for upregulated genes in Brca2mut/WT P4 HU cluster 3
platelet degranulation
neutrophil degranulation
neutrophil activation involved in immune response
neutrophil mediated immunity
negative regulation of viral process
log10pval
4.95
4.90
4.85
4.80
4.75
4.70
0
5
−log10(pval)
E)
MisgDB
Cluster 1
KRAS Signaling Up
Estrogen Response Late
TGF−beta Signaling*
Cholesterol Homeostasis*
IL−6/JAK/STAT3 Signaling*
log10pval
2.00
1.75
1.50
0
5
−log10(pval)
Cluster 2
Interferon Gamma Response
Interferon Alpha Response
Epithelial Mesenchymal Transition
Coagulation
Estrogen Response Late
log10pval
8
6
4
0
5
10
−log10(pval)
Cluster 3
Epithelial Mesenchymal Transition
Coagulation
Apoptosis
Cholesterol Homeostasis
IL−2/STAT5 Signaling
log10pval
9
8
7
6
5
4
3
0
5
10
−log10(pval)
Cluster 4
Interferon Gamma Response
Interferon Alpha Response
TNF−alpha Signaling via NF−kB
p53Pathway
Hypoxia
log10pval
20
15
10
0
5
10
15
20
25
−log10(pval)
* - not significant
F)
Wildtype_C_P0
Wildtype_C_P4
Wildtype_HU_P0
Wildtype_HU_P4
Brca2mut/WT_C_P0
Brca2mut/WT_C_P4
Brca2mut/WT_HU_P4
Brca2mut/WT_HU_P4
Pdr3a
Pdr3b
Pdr3c
Pdr3d
Pdr3e
Pdr1c
Pdr3k
Pdr1d
Pdr3h
Pdr3g
Pdr3gl
Pdr3f
Pdr2e
Pdr2f
Pdr2h
Pdr2k
Pdr2l
Mavs
Nfkb1
Rela
Il6
Cgas
Tbk1
Ikbke
Irf3
Irf7
Ifnb1
Zbp1
Ripk1
Ripk3
Ikbkg
Chuk
Ikbkb
Nfkbib
Nfkbia
Ccl4
Ccl5
Cxcl10
Aim2
Fadd
Casp8
Pycard
Casp1
Mlkl
Casp3
Casp7
Gsdme
Gsdmd
Il18
Il33
Adar
Samhd1
Dnase2a
Ifi202b
Expression
2
1
0
−1
G)
KEGG
Cluster 4
Epstein−Barr virus infection
Antigen processing and presentation
Hepes simplex virus 1 infection
Viral carcinogenesis
Human immunodeficiency virus 1 infection
log10pval
14
12
10
8
0
5
10
15
−log10(pval)
H)
GO for upregulated genes in Wildtype-HU Cluster 5
maturation of LSU−rRNA
mitotic cell cycle phase transition
DNA damage response signal transduction by p53 class mediator resulting in cell cycle arrest
ribosomal large subunit biogenesis
mitotic G1 DNA damage checkpoint signaling
0
5
10
15
−log10(pval)
I)
GO for upregulated genes in Widltype-C Cluster 1
regulation of receptor−mediated endocytosis
positive regulation of receptor−mediated endocytosis
positive regulation of endocytosis
neutrophil degranulation
neutrophil activation involved in immune response
log10pval
3.20
3.15
3.10
3.05
0
5
10
−log10(pval)
J)
GO for upregulated genes in Wildtype-C Cluster 4
neutrophil degranulation
neutrophil activation involved in immune response
neutrophil mediated immunity
cellular iron ion homeostasis
neuron apoptotic process
log10pval
8
7
6
5
0
5
10
−log10(pval)

Supplementary Figure 7**. Single-cell transcriptomics of HR- luminal cells from Brca2$^{mut/WT}$ and wildtype organoids.** A) Frequency of HR- luminal cell clusters for each group, colour coded by cluster. The top 5 GO Biological terms associated with the upregulated genes in Brca2$^{mut/WT}$ HU passage 4 of B) Cluster 1, C) Cluster 2 and D) Cluster 3 cells. E) The top 5 MSigDB Hallmark terms associated with the upregulated genes in Brca2$^{mut/WT}$ HU passage 4 of Cluster 1, Cluster 2, Cluster 3 and Cluster 4 cells. F) Heatmap of the genes detailed in the Cytosolic DNA-sensing pathway from mmu04623 KEGG from all organoid groups. G) The top 5 KEGG terms associated with the upregulated genes in Brca2$^{mut/WT}$ HU passage 4 of Cluster 4. H) The top 5 GO Biological terms associated with the upregulated genes in Wildtype HU passage 4 compared with only the Wildtype control cells of Cluster 5. The top 5 GO Biological terms associated with the upregulated genes in Wildtype control passage 4 cells of I) Cluster 1 and J) Cluster 4.

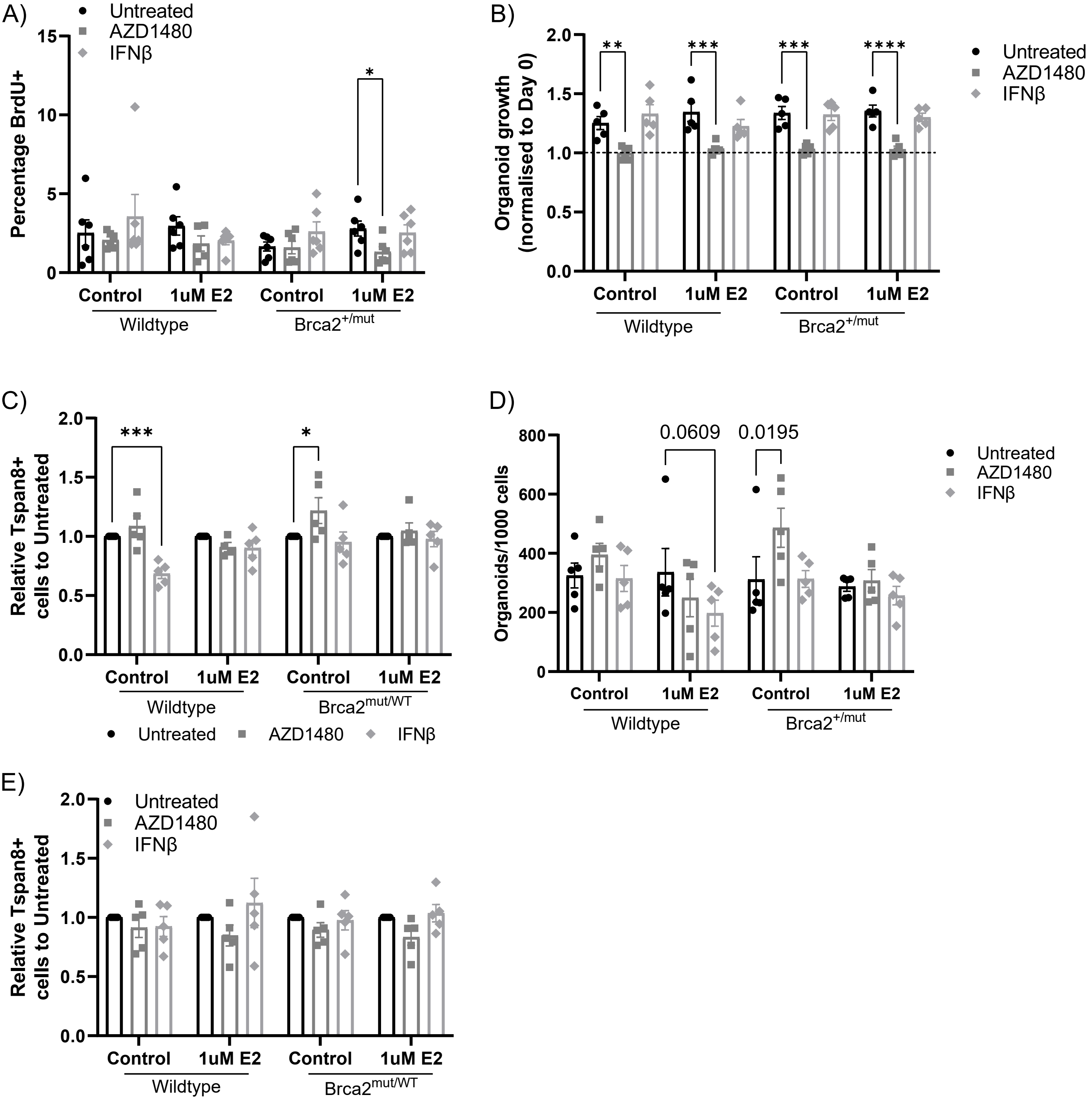

A)
Untreated
AZD1480
IFNβ
Percentage BrdU+
15
10
5
0
*
Control
1uM E2
Control
1uM E2
Wildtype
Brca2+/mut
B)
Organoid growth (normalised to Day 0)
2.0
1.5
1.0
0.5
0.0
**
***
***
****
Untreated
AZD1480
IFNβ
Control
1uM E2
Control
1uM E2
Wildtype
Brca2+/mut
C)
Relative Tspan8+ cells to Untreated
2.0
1.5
1.0
0.5
0.0
***
*
Control
1uM E2
Control
1uM E2
Wildtype
Brca2mut/WT
Untreated
AZD1480
IFNβ
D)
Organoids/1000 cells
800
600
400
200
0
0.0609
0.0195
Untreated
AZD1480
IFNβ
Control
1uM E2
Control
1uM E2
Wildtype
Brca2+/mut
E)
Relative Tspan8+ cells to Untreated
2.0
1.5
1.0
0.5
0.0
Untreated
AZD1480
IFNβ
Control
1uM E2
Control
1uM E2
Wildtype
Brca2mut/WT

Supplementary Figure 8. **Type I interferon response are not activated following Estrogenic stresses.** A) Quantification of the percentage of BrdU+ cells in the HR- luminal subpopulations from wildtype or $Brca2^{mut/WT}$ passage 4 organoids after 4 days of IFNβ or AZD1480 treatment in control and E2 exposed organoids. Data presented as mean +/- SEM (n=5). An ANOVA followed by a Fishers LSD test was performed, *p=0.0411. Source data provided. B) Quantification of average organoid size after 4 days of INFβ or AZD1480 treatment normalised to Day 0 treatment organoid size in control and E2 exposed organoids. Dashed line indicates Day 0 area. Data presented as mean +/- SEM (n=5). An ANOVA followed by a Dunnett's multiple comparison test, **p=0.0015 and ***p<0.0002. Source data provided. C) Quantification of the percentage of Tspan8+ cells in the HR- luminal population after 4 days of IFNβ or AZD1480 treatment on passage 4 wildtype or $Brca2^{mut/WT}$ organoids in control and E2 exposed organoids. Data presented as the mean +/- SEM (n=5). An ANOVA followed by a Fishers LSD test, * p = 0.013, *** p = 0.0005. Source data provided. D) Quantification of the number of organoids formed from wildtype or $Brca2^{mut/WT}$ passage 4 single cells after IFNβ or AZD1480 treatments in control and E2 exposed organoids. Data presented as mean +/- SEM (n=5). An ANOVA followed by a Fishers LSD test was performed. Source data provided. E) Quantification of the percentage of Tspan8 + cells in the HR- luminal populations of wildtype or $Brca2^{mut/WT}$ organoids from D). Data presented as the mean +/- SEM (n=5). An ANOVA followed by a Fishers LSD test showed no statistical differences. Source data are provided as a Source Data file.

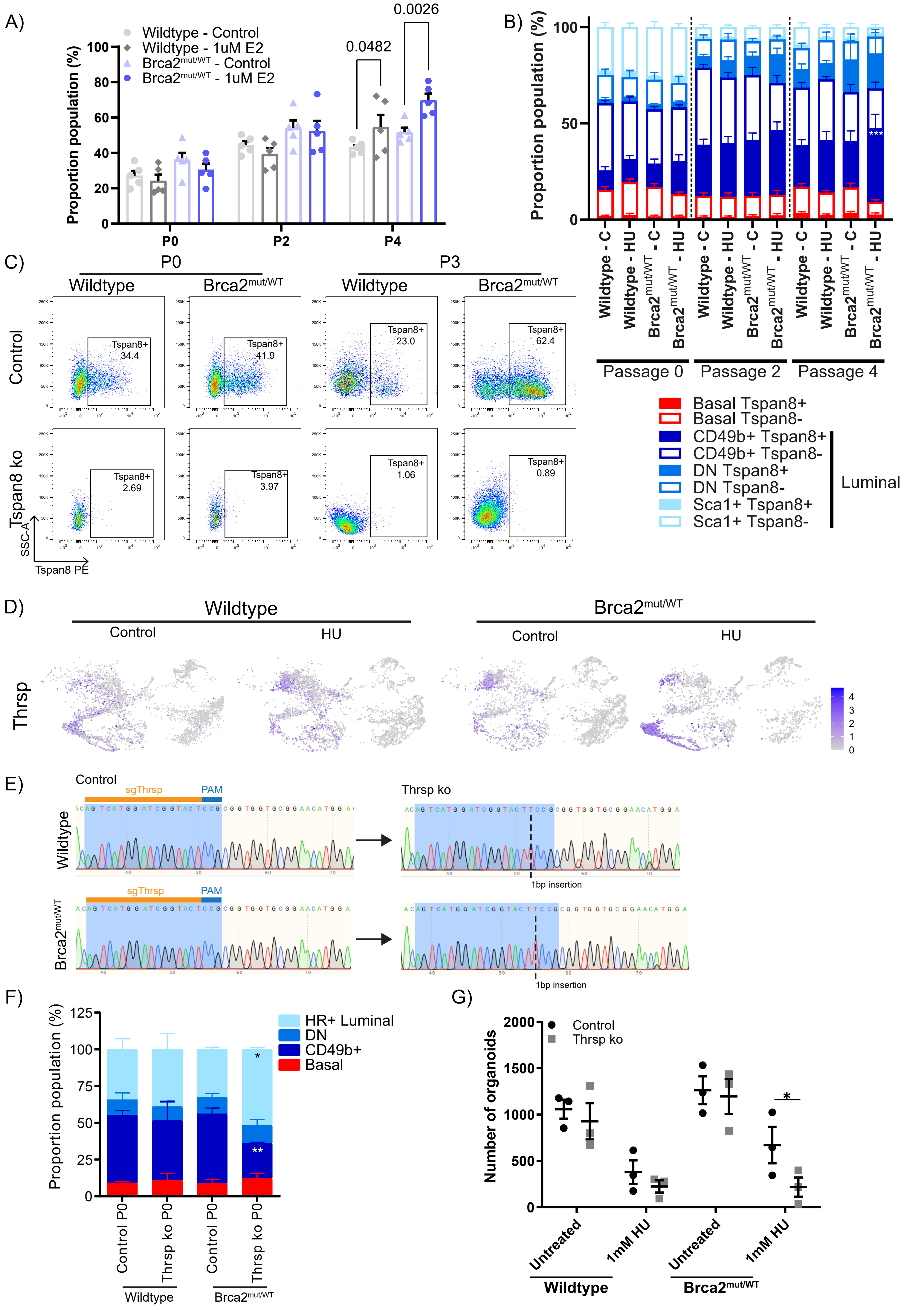
A)
Wildtype - Control
Wildtype - 1uM E2
Brca2mut/WT - Control
Brca2mut/WT - 1uM E2
Proportion population (%)
0.0482
0.0026
P0
P2
P4
B)
Proportion population (%)
Wildtype - C
Wildtype - HU
Brca2mut/WT - C
Brca2mut/WT - HU
Passage 0
Passage 2
Passage 4
Basal Tspan8+
Basal Tspan8-
CD49b+ Tspan8+
CD49b+ Tspan8-
DN Tspan8+
DN Tspan8-
Sca1+ Tspan8+
Sca1+ Tspan8-
Luminal
C)
P0
P3
Wildtype
Brca2mut/WT
Control
Tspan8 ko
Tspan8+ 34.4
Tspan8+ 41.9
Tspan8+ 23.0
Tspan8+ 62.4
Tspan8+ 2.69
Tspan8+ 3.97
Tspan8+ 1.06
Tspan8+ 0.89
SSC-A
Tspan8 PE
D)
Wildtype
Brca2mut/WT
Control
HU
Thrsp
E)
Control
sgThrsp
PAM
Thrsp ko
Wildtype
Brca2mut/WT
1bp insertion
F)
Proportion population (%)
HR+ Luminal
DN
CD49b+
Basal
Control P0
Thrsp ko P0
Wildtype
Brca2mut/WT
G)
Number of organoids
Control
Thrsp ko
Untreated
1mM HU
Wildtype
Brca2mut/WT

Supplementary Figure 9. **Deletion of Thrsp expression in the HR- luminal cells render Brca2$^{mut/WT}$ organoids incapable of recovery from DNA damage.** A) Quantification of the percentage of Tspan8+ cells in the luminal progenitor population over different passages (P0, P2 and P4) from wildtype or Brca2$^{mut/WT}$ organoids treated with E2. Data presented as the mean +/- SD (n=5). An ANOVA followed by a Fishers LSD test was performed. Source data provided. B) Quantification of the percentage of Tspan8+ cells in the epithelial populations over different passages (P0, P2, and P4) from wildtype or Brca2$^{mut/WT}$ organoids treated with HU. Data presented as the mean +/- SD (n=7). An ANOVA followed by a Fishers LSD test was performed. ***p=0.0003. C) Representative flow cytometry analysis of Tspan8 expression from control (upper) or Tspan8 knockout (ko, lower) wildtype and Brca2$^{mut/WT}$ organoids at passage 0 and passage 3. D) UMAP with overlaid expression of *Thrsp* gene from control or HU treated passage 4 wildtype or Brca2$^{mut/WT}$ organoids. E) Illustration of Sanger sequencing results for the *Thrsp* indel created by CRISPR/Cas9 in wildtype and Brca2$^{mut/WT}$ mammary cells. F) Quantification of the percentage of epithelial cell types from control or Thrsp ko wildtype or Brca2$^{mut/WT}$ organoids. Data presented mean +/- SD (n=2/3). An ANOVA followed by a Fishers LSD test, *p=0.0225, **p=0.0067. Source data provided. G) Quantification of the number of wildtype or Brca2$^{mut/WT}$ organoids from control and Thrsp ko post HU treatments at passage 3. Data presented mean +/- SEM (n=3). An ANOVA followed by a Fishers LSD test, *p=0.0463. Source data are provided as a Source Data file.

A)

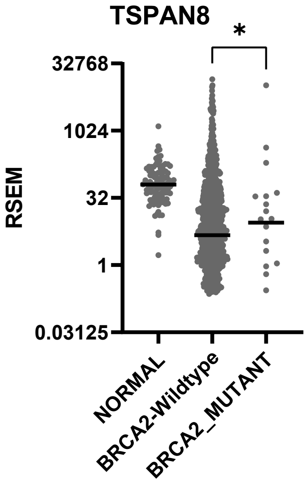
TSPAN8
*
32768
1024
32
1
0.03125
RSEM
NORMAL
BRCA2-Wildtype
BRCA2_MUTANT

B)

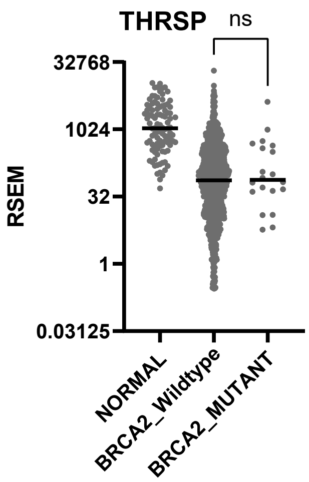
THRSP
ns
32768
1024
32
1
0.03125
RSEM
NORMAL
BRCA2_Wildtype
BRCA2_MUTANT

C)

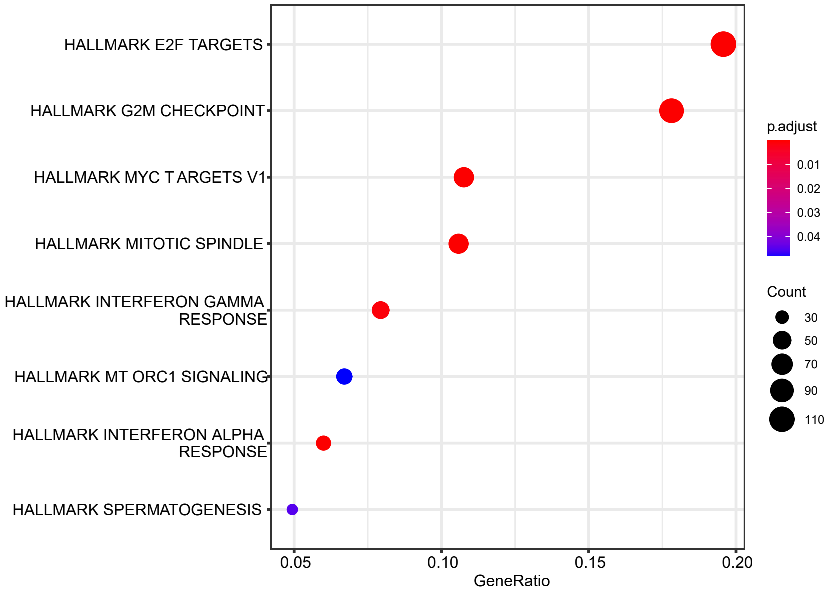
HALLMARK E2F TARGETS
HALLMARK G2M CHECKPOINT
HALLMARK MYC T ARGETS V1
HALLMARK MITOTIC SPINDLE
HALLMARK INTERFERON GAMMA RESPONSE
HALLMARK MT ORC1 SIGNALING
HALLMARK INTERFERON ALPHA RESPONSE
HALLMARK SPERMATOGENESIS
0.05
0.10
0.15
0.20
GeneRatio
p.adjust
0.01
0.02
0.03
0.04
Count
30
50
70
90
110

Supplementary Figure 10. **Human BRCA2 tumour expression and interferon pathway analysis.** Expression levels of A) TSPAN8 and B) THRSP in normal tissues, non-BRCA2 carriers (BRCA2-Wildtype) or germline BRCA2 carriers (BRCA2-MUTANT) from the TCGA dataset. An unpaired t-Test was performed. * p = 0.0405. RSEM = RNA-Seq by Expectation-Maximization. C) A dot plot of upregulated pathways from BRCA2 mutant tumours from the TCGA dataset.

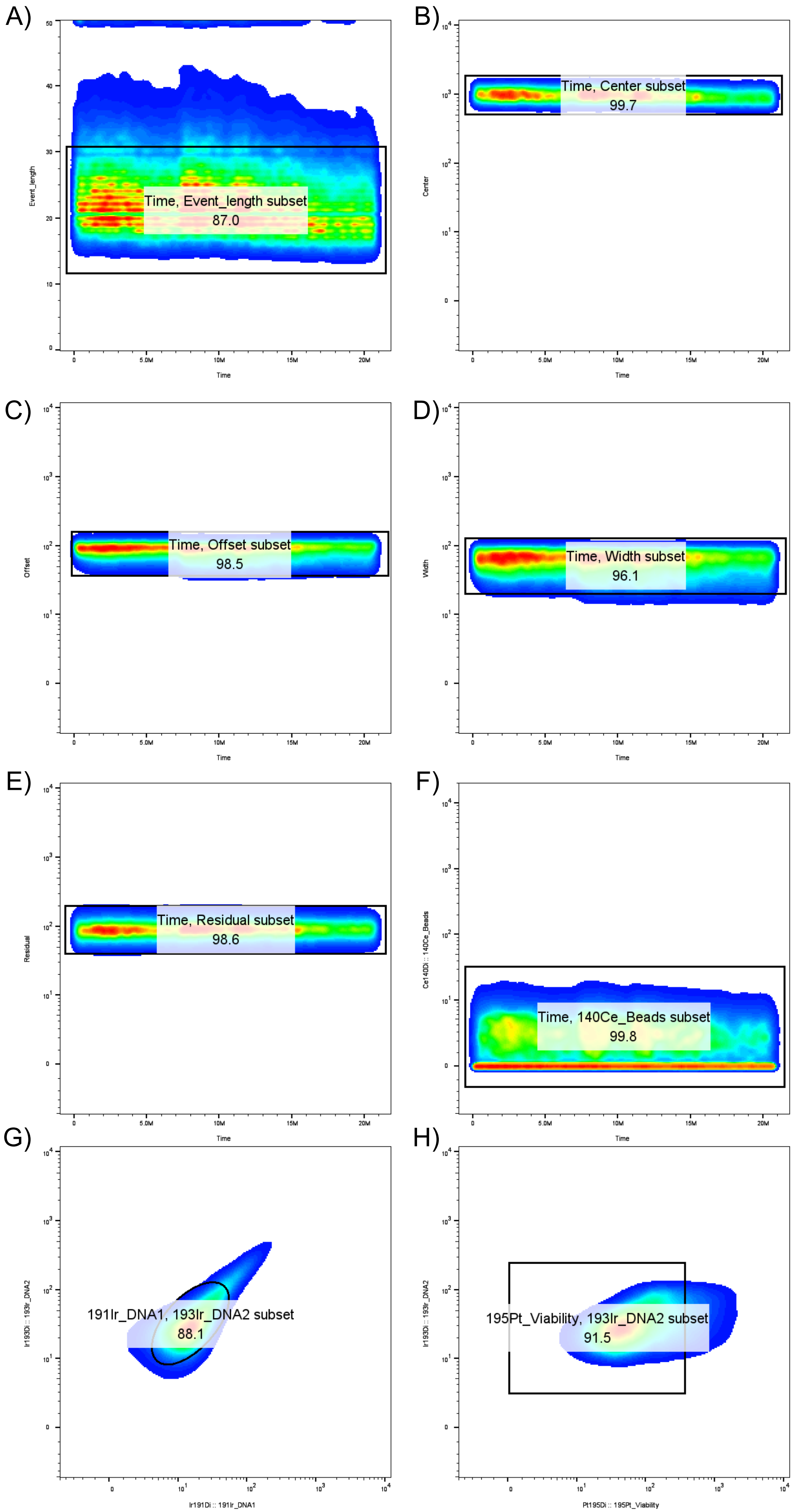

A)
Time, Event_length subset
87.0
Event_length
Time
B)
Time, Center subset
99.7
Center
Time
C)
Time, Offset subset
98.5
Offset
Time
D)
Time, Width subset
96.1
Width
Time
E)
Time, Residual subset
98.6
Residual
Time
F)
Time, 140Ce_Beads subset
99.8
Ce140Di :: 140Ce_Beads
Time
G)
191Ir_DNA1, 193Ir_DNA2 subset
88.1
Ir193Di :: 193Ir_DNA2
Ir191Di :: 191Ir_DNA1
H)
195Pt_Viability, 193Ir_DNA2 subset
91.5
Ir193Di :: 193Ir_DNA2
Pt195Di :: 195Pt_Viability

Supplementary Figure 11. **Flow gating strategy for CyTOF.** A) Time Event length B) Time Centre subset, C) Time Offset subset, D) Time Width subset, E) Time Residual subset, F) Time 140Ce subset, G) DNA content 191Ir, DNA content 193Ir and H) Viability 195Pt.

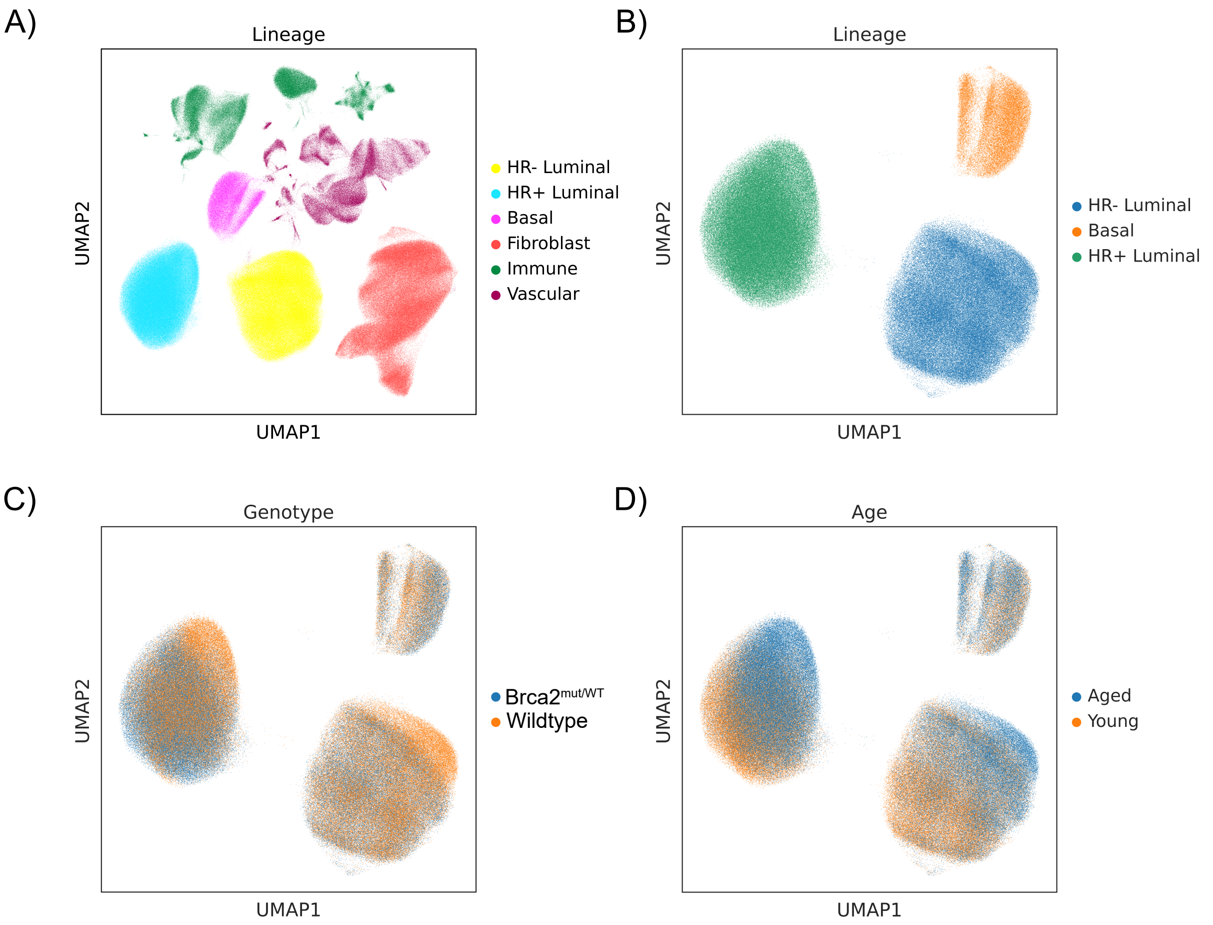
A)
Lineage
HR- Luminal
HR+ Luminal
Basal
Fibroblast
Immune
Vascular
UMAP1
UMAP2
B)
Lineage
HR- Luminal
Basal
HR+ Luminal
UMAP1
UMAP2
C)
Genotype
Brca2mut/WT
Wildtype
UMAP1
UMAP2
D)
Age
Aged
Young
UMAP1
UMAP2

Supplementary Figure 12. **UMAPs of mammary epithelial cells using CyTOF.** A) UMAP of all mammar y cells in which each dot represents one cell. Cells are coloured by lineage. B) UMAP of the epithelial l ineages, coloured by lineage type. C) UMAP of epithelial lineages, coloured by genotype. D) UMAP of epithelial lineages, coloured by age.

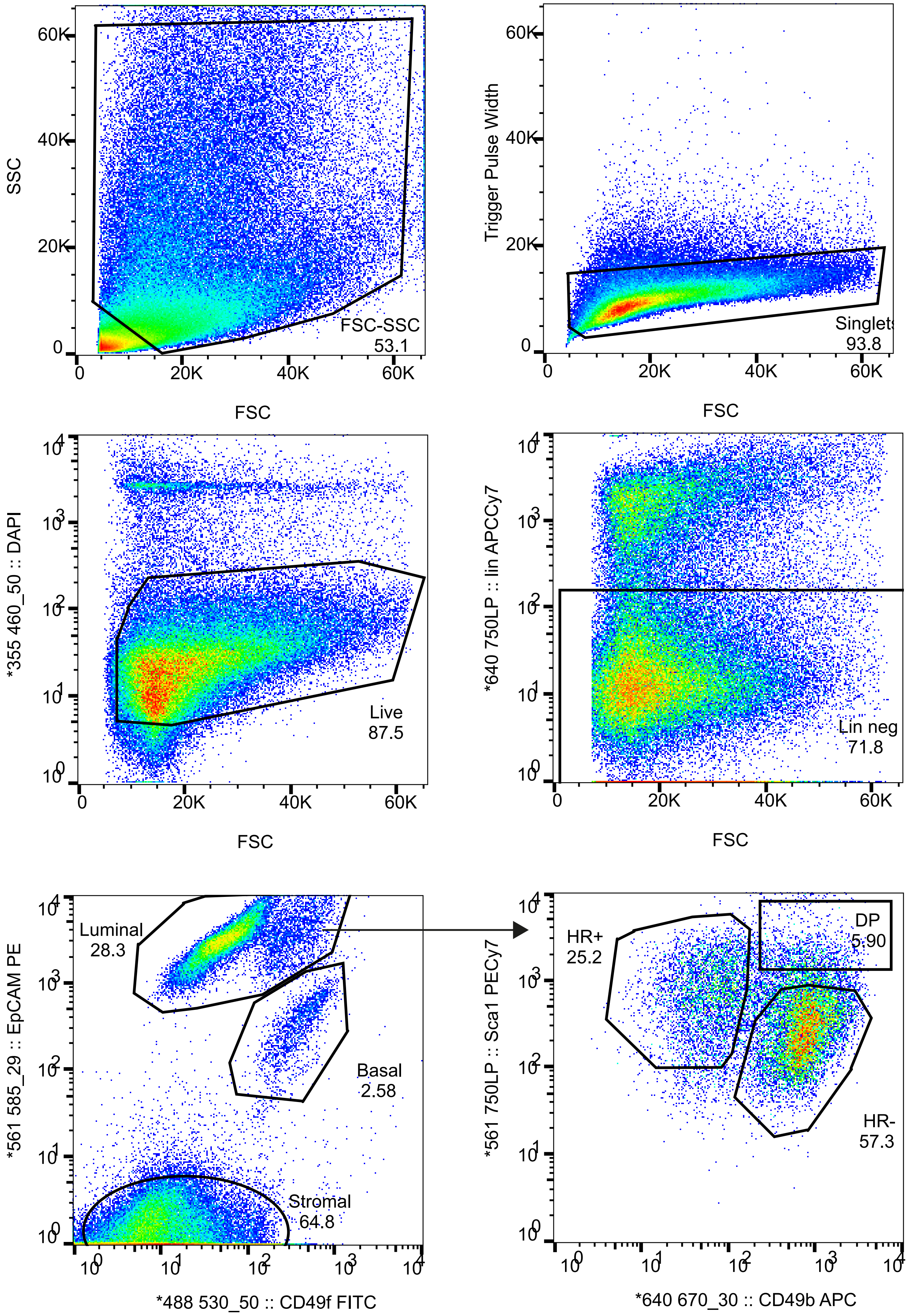

SSC
FSC
FSC-SSC
53.1
Trigger Pulse Width
Singlets
93.8
*355 460_50 :: DAPI
Live
87.5
*640 750LP :: lin APCCy7
Lin neg
71.8
*561 585_29 :: EpCAM PE
*488 530_50 :: CD49f FITC
Luminal
28.3
Basal
2.58
Stromal
64.8
*561 750LP :: Sca1 PECy7
*640 670_30 :: CD49b APC
HR+
25.2
DP
5.90
HR-
57.3

Supplementary Figure 13. **Gating strategy for flow cytometric analysis and sorting for mouse mammary epithelial cells.** Cells were gated on forward (FSC) and side (SSC) scatter to remove debris. Then trigger pulse width to obtain single cells. 4',6-diamidino-2-phenylindole (DAPI)-positive and lineage-positive cells were excluded. Cells were then identified to be luminal, basal or stromal based on the EpCAM and CD49f expression, and regions drawn. Luminal cells were then further fractioned based on Sca1 and CD49b expression, with regions drawn to identify the three populations.

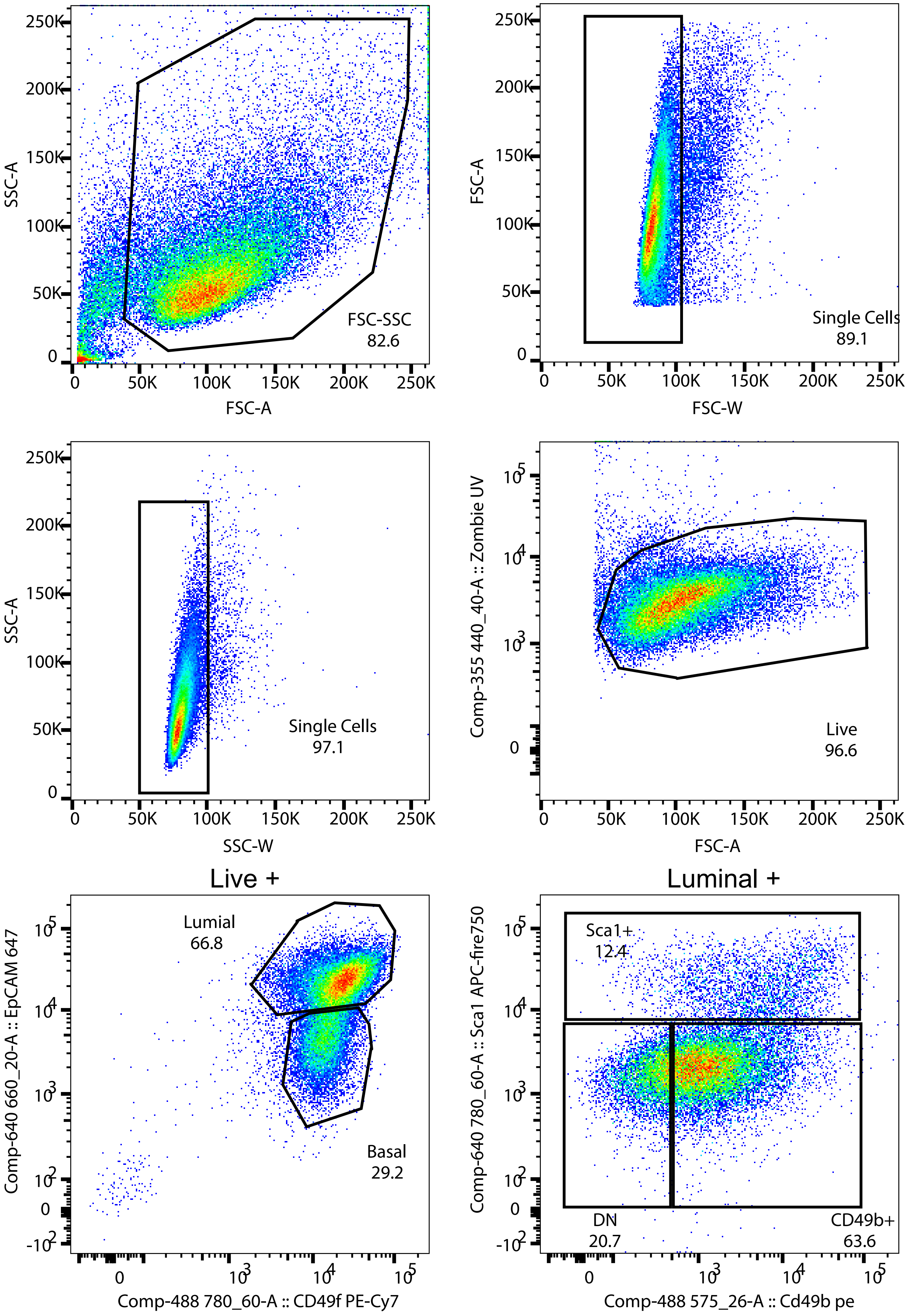
SSC-A
FSC-A
FSC-SSC
82.6
FSC-A
FSC-W
Single Cells
89.1
SSC-A
SSC-W
Single Cells
97.1
Comp-355 440_40-A :: Zombie UV
FSC-A
Live
96.6
Live +
Comp-640 660_20-A :: EpCAM 647
Comp-488 780_60-A :: CD49f PE-Cy7
Lumial
66.8
Basal
29.2
Luminal +
Comp-640 780_60-A :: Sca1 APC-fire750
Comp-488 575_26-A :: Cd49b pe
Sca1+
12.4
DN
20.7
CD49b+
63.6

Supplementary Figure 14. **Gating strategy for flow cytometric analysis of mammary organoid cultures.** Cells were gated on FSC and SSC scatter to remove debris. Then FSC-W/A and SSC-W/A were selected respectively to obtain single cells. Zombie UV-positive cells were excluded. Live positive cells were then identified to be luminal or basal based on the EpCAM and CD49f expression, and regions drawn. Luminal positive cells were then further fractioned based on Sca1 and CD49b expression, with regions drawn to identify the three populations.

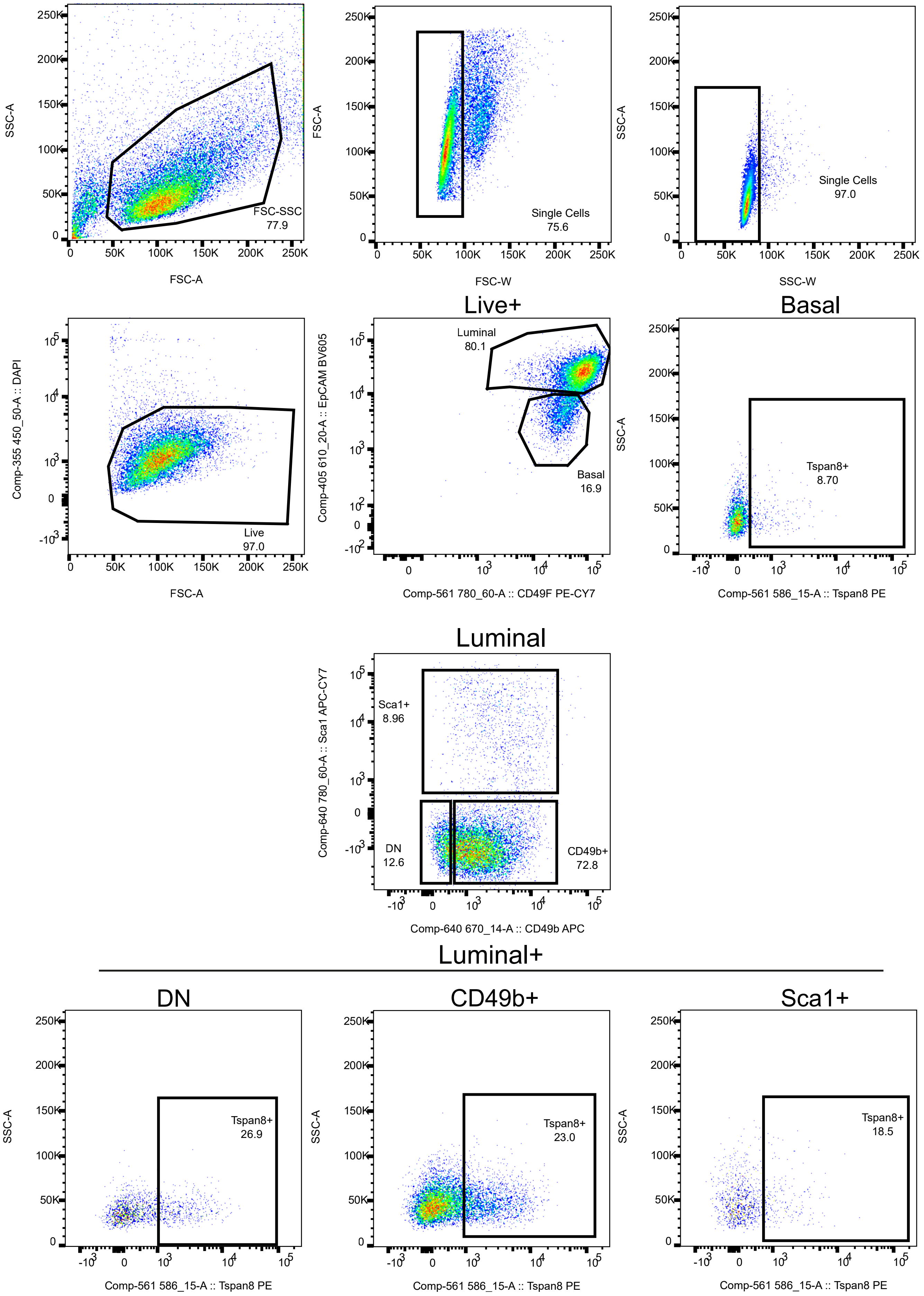
FSC-SSC
77.9
SSC-A
FSC-A
Single Cells
75.6
FSC-A
FSC-W
Single Cells
97.0
SSC-A
SSC-W
Live
97.0
Comp-355 450_50-A :: DAPI
FSC-A
Live+
Luminal
80.1
Basal
16.9
Comp-405 610_20-A :: EpCAM BV605
Comp-561 780_60-A :: CD49F PE-CY7
Basal
Tspan8+
8.70
SSC-A
Comp-561 586_15-A :: Tspan8 PE
Luminal
Sca1+
8.96
DN
12.6
CD49b+
72.8
Comp-640 780_60-A :: Sca1 APC-CY7
Comp-640 670_14-A :: CD49b APC
Luminal+
DN
Tspan8+
26.9
CD49b+
Tspan8+
23.0
Sca1+
Tspan8+
18.5
SSC-A
Comp-561 586_15-A :: Tspan8 PE

Supplementary Figure 15. **Gating strategy for Tspan8 flow cytometric analysis of mammary organoid cultures.** Cells were gated on FSC and SSC scatter to remove debris. Then FSC-W/A and SSC-W/A were selected respectively to obtain single cells. Zombie UV-positive cells were excluded. Live positive cells were then identified to be luminal or basal based on the EpCAM and CD49f expression, a nd regions drawn. Luminal positive cells were then further fractioned based on Sca1 and CD49b expression, with regions drawn to identify the three populations. Tspan8 positive cells were then gated for each epithelial subpopulation.

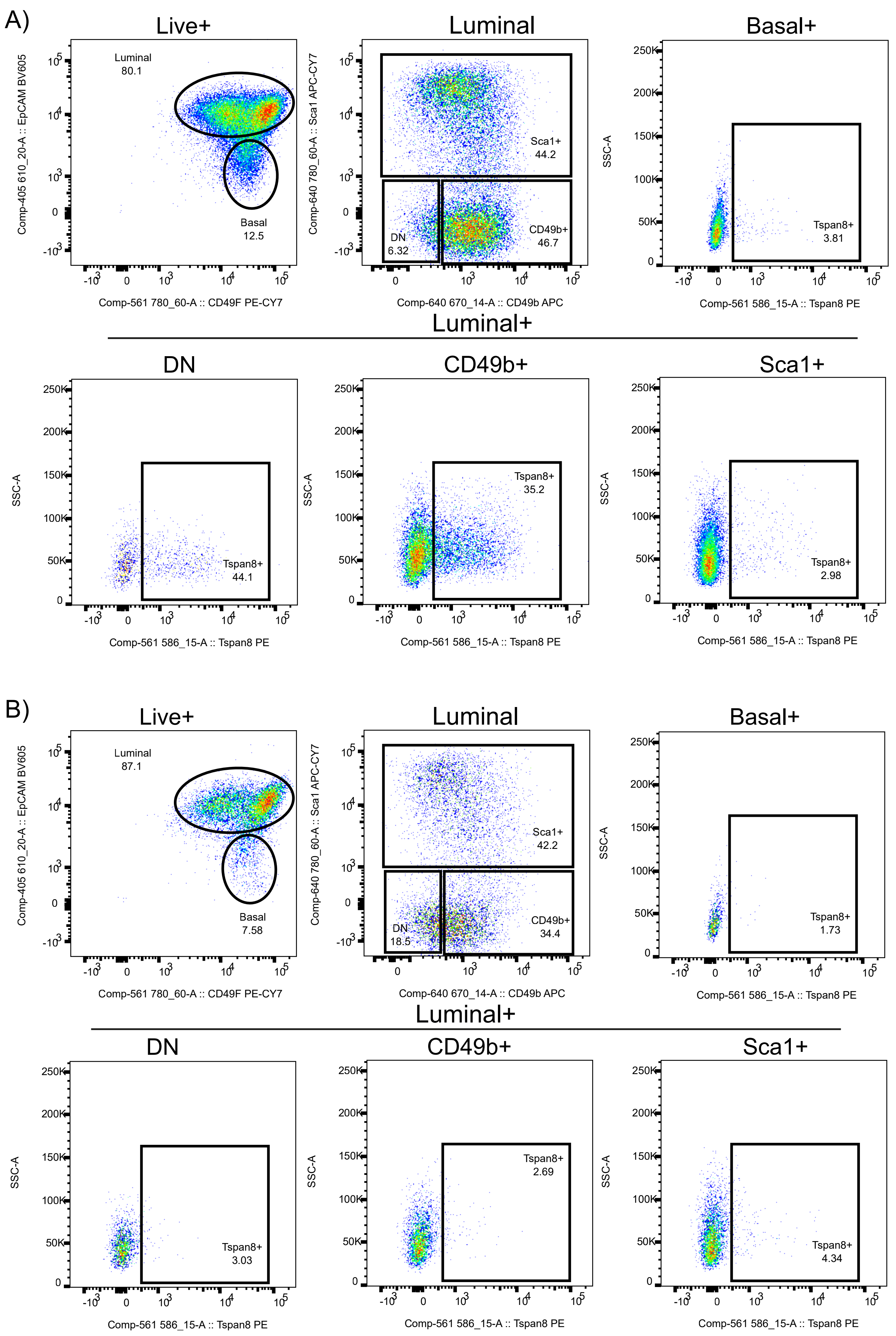

A)
Live+
Luminal
80.1
Basal
12.5
Comp-405 610_20-A :: EpCAM BV605
Comp-561 780_60-A :: CD49F PE-CY7
Luminal
Sca1+
44.2
DN
6.32
CD49b+
46.7
Comp-640 780_60-A :: Sca1 APC-CY7
Comp-640 670_14-A :: CD49b APC
Basal+
Tspan8+
3.81
SSC-A
Comp-561 586_15-A :: Tspan8 PE
Luminal+
DN
Tspan8+
44.1
CD49b+
Tspan8+
35.2
Sca1+
Tspan8+
2.98
B)
Live+
Luminal
87.1
Basal
7.58
Luminal
Sca1+
42.2
DN
18.5
CD49b+
34.4
Basal+
Tspan8+
1.73
Luminal+
DN
Tspan8+
3.03
CD49b+
Tspan8+
2.69
Sca1+
Tspan8+
4.34

Supplementary Figure 16. **Gating strategy for Tspan8 CRISPR.** Live positive cells were identified to be luminal or basal based on the EpCAM and CD49f expression, and regions drawn. Luminal positive cells were then further fractioned based on Sca1 and CD49b expression, with regions drawn to identify the three populations. Tspan8 positive cells were then gated for each epithelial subpopulation. Representative mammary epithelial sample depicting the gating strategy for A) untargeted and B) Tspan8 CRISPR edited cells.

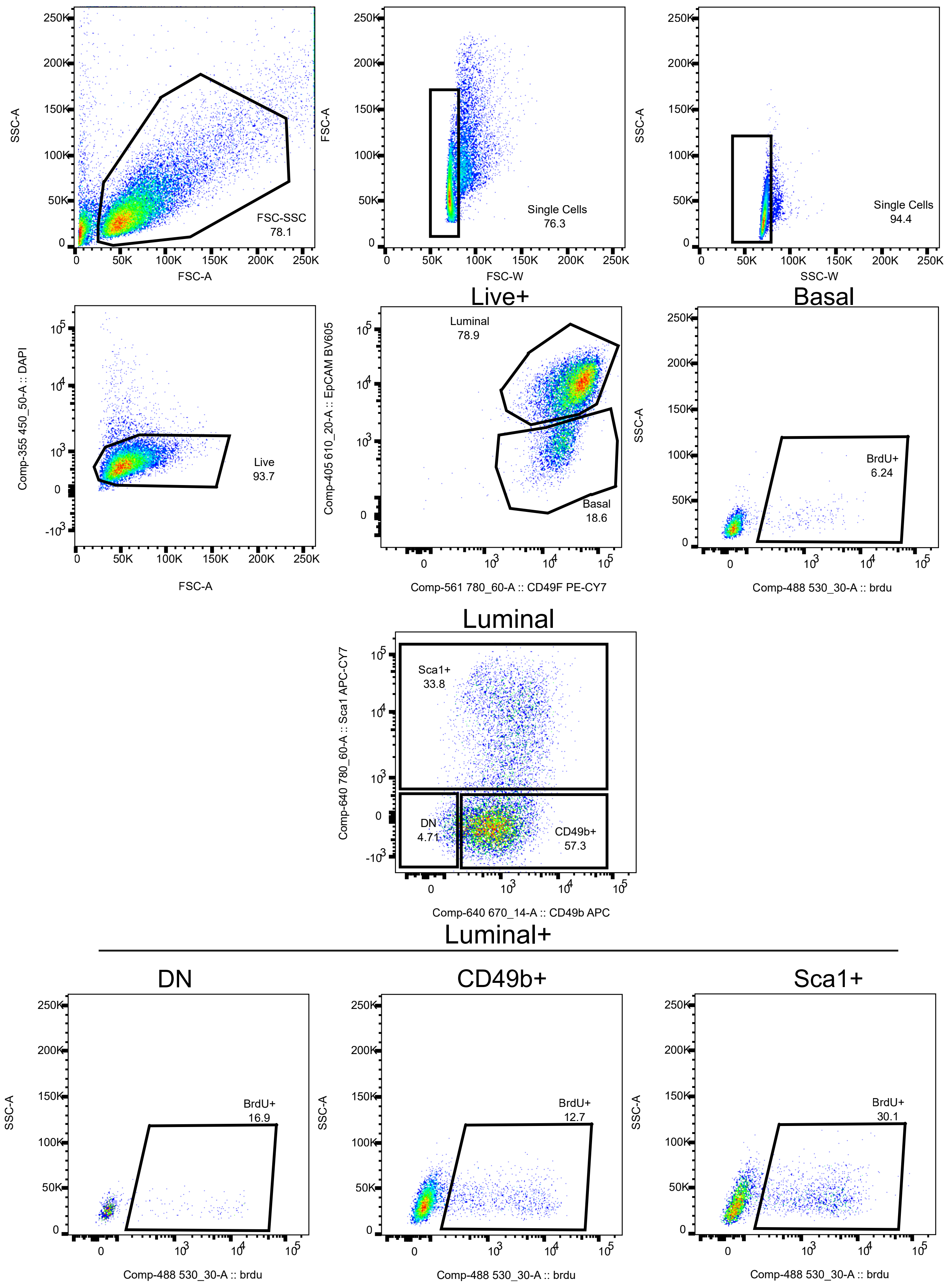
SSC-A
FSC-A
FSC-SSC
78.1
FSC-W
Single Cells
76.3
SSC-W
Single Cells
94.4
Live+
Basal
Comp-355 450_50-A :: DAPI
Live
93.7
Comp-405 610_20-A :: EpCAM BV605
Luminal
78.9
Basal
18.6
Comp-561 780_60-A :: CD49F PE-CY7
BrdU+
6.24
Comp-488 530_30-A :: brdu
Luminal
Comp-640 780_60-A :: Sca1 APC-CY7
Sca1+
33.8
DN
4.71
CD49b+
57.3
Comp-640 670_14-A :: CD49b APC
Luminal+
DN
CD49b+
Sca1+
BrdU+
16.9
BrdU+
12.7
BrdU+
30.1

Supplementary Figure 17. **Gating strategy for BrdU flow cytometric analysis of mammary organoid cultures.** Cells were gated on FSC and SSC scatter to remove debris. Then FSC-W/A and SSC-W/A were selected respectively to obtain single cells. Zombie UV-positive cells were excluded. Live positive cells were then identified to be luminal or basal based on the EpCAM and CD49f expression, and regions drawn. Luminal positive cells were then further fractioned based on Sca1 and CD49b expression, with regions drawn to identify the three populations. BrdU positive cells were then gated for each epithelial subpopulation.

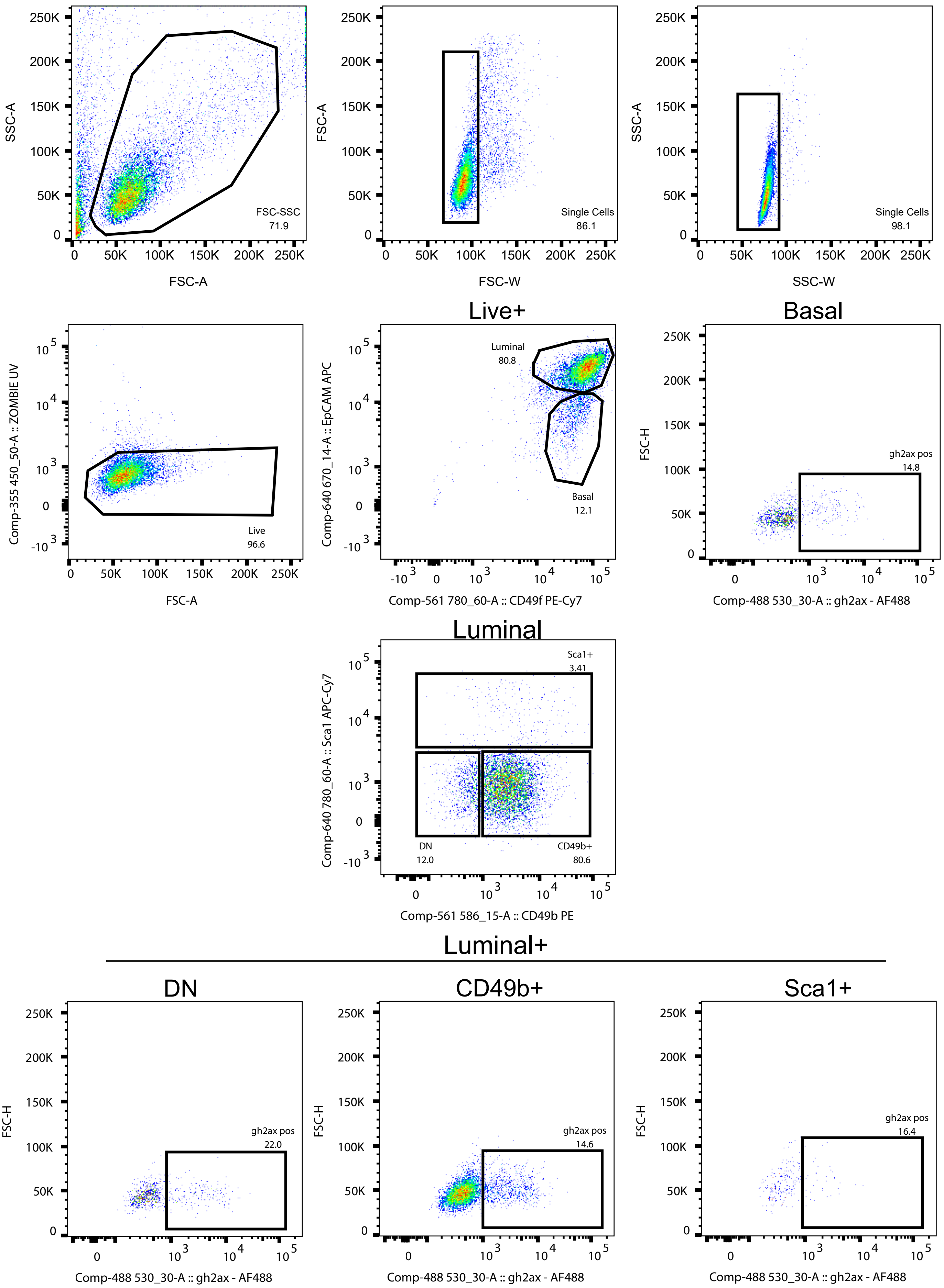
FSC-SSC
71.9
SSC-A
FSC-A
Single Cells
86.1
FSC-A
FSC-W
Single Cells
98.1
SSC-A
SSC-W
Live
96.6
Comp-355 450_50-A :: ZOMBIE UV
FSC-A
Live+
Luminal
80.8
Basal
12.1
Comp-640 670_14-A :: EpCAM APC
Comp-561 780_60-A :: CD49f PE-Cy7
Basal
gh2ax pos
14.8
FSC-H
Comp-488 530_30-A :: gh2ax - AF488
Luminal
Sca1+
3.41
DN
12.0
CD49b+
80.6
Comp-640 780_60-A :: Sca1 APC-Cy7
Comp-561 586_15-A :: CD49b PE
Luminal+
DN
gh2ax pos
22.0
CD49b+
gh2ax pos
14.6
Sca1+
gh2ax pos
16.4
FSC-H
Comp-488 530_30-A :: gh2ax - AF488

Supplementary Figure 18. **Gating strategy for γH2AX flow cytometric analysis of mammary organoid cultures.** Cells were gated on FSC and SSC scatter to remove debris. Then FSC-W/A and SSC-W/A were selected respectively to obtain single cells. Zombie UV-positive cells were excluded. Live positive cells were then identified to be luminal or basal based on the EpCAM and CD49f expression, and regions drawn. Luminal positive cells were then further fractioned based on Sca1 and CD49b expression, with regions drawn to identify the three populations. γH2AX positive cells were then gated for each epithelial subpopulation.

Supplementary Table 1: Primary antibodies.

Epitope	Host	Dilution	Catalog number	Supplier
Tissue Sections:				
Keratin 14	Chicken	1/1000	906004	BioLegend
Progesterone Receptor	Mouse	1/100	66300-1-IG	Proteintech
CD36	Rabbit	1/250	18836-1-AP	Proteintech
CD14	Mouse	1/100	60253-1-Ig	Proteintech
E-Cadherin	Rabbit	1/500	20874-1-AP	Proteintech
Wholemounts:				
Keratin 14	Chicken	1/1000	906004	BioLegend
Progesterone Receptor	Mouse	1/100	66300-1-IG	Proteintech
E-Cadherin	Mouse	1/500	610181	BD
Vimentin	Rabbit	1/1000	ab92547	Abcam

Supplementary Table 2 Secondary antibodies.

Host	Species Reactivity	Conjugation	Dilution	Catalog number	Supplier
Goat	Chicken	AF488	1/500	103-545-155	Jackson ImmnunoResearch
Goat	Mouse	AF647	1/500	115-545-166	Jackson ImmnunoResearch
Goat	Rabbit	Cy3	1/500	111-165-003	Jackson ImmnunoResearch

Supplementary Table 3: Antibodies used for flow cytometry

Epitope [clone]	Conjugation	Dilution	Catalog number	Supplier
EpCAM [G8.8]	Brilliant Violet 605	1/400	118227	BioLegend
CD49f [GoH3]	PE/Cy7	1/400	313622	BioLegend
CD49b [HMa2]	APC	1/100	103515	BioLegend
Sca1 [D7]	APC/Fire750	1/1000	108145	BioLegend
CD31 [390]	Biotin	1/500	13-0311-81	Invitrogen
CD45 [30-F11]	Biotin	1/500	47-0451-80	Invitrogen
Ter119 [TER119]	Biotin	1/500	116204	BioLegend
Tspan8 [657909]	PE	1/100	FAB6524P	R&D Systems
BrdU [3D4]	FITC	1/100	364105	BioLegend
Phospho-Histone H2A.X (Ser139) [20E3]	FITC	1/200	9719S	Cell Signalling Technologies

Supplementary Table 4: Antibodies used for CyTOF mass cytometry

Epitope [clone]	Metal	Dilution	Catalog number	Supplier
TER119 [Ly-76]	113In	1/100	116201	Biolegend
CD31 [MEC13.3]	115In	1/100	102502	Biolegend
PCAD [Polyclonal]	141Pr	1/100	AF761	R&D Systems
SSEA4 [MC-813-70]	142Nd	1/100	330402	Biolegend
Procr [eBio1560]	143Nd	1/100	16-2012-83	EBioScience
CD200 [OX-90]	144Nd	1/100	123802	Biolegend
Sca1 [E13-161.7]	145Nd	1/100	122502	Biolegend
EGFR [D1D4J]	146Nd	1/100	54359S	Cell Signaling
Kit [2B8]	147Sm	1/100	105802	Biolegend
CD49f [GoH3]	148Nd	1/100	313602	Biolegend
CD9 [MZ3]	149Sm	1/100	124802	Biolegend
CD44 [IM7]	150Nd	1/100	103002	Biolegend
CD61 [2C9.G2]	151Eu	1/100	104302	Biolegend
ALCAM [eBioALC48]	152Sm	1/100	14-1661-82	EBioScience
CD36 [HM36]	154SM	1/100	102602	Biolegend
CD66a [Mab-CC1]	153Eu	1/100	134506	Biolegend
ECAD [Polyclonal]	155Gd	1/100	AF748	R&D Systems
Tspan8 [657909]	156Gd	1/100	MAB6524	R&D Systems
CD24 [M1/69]	158Gd	1/100	11-0242-85	EBioScience
CD49b [HMalpha2]	159Tb	1/100	103501	Biolegend
Ly6D [49-H4]	160Gd	1/100	557360	BD Bioscience
CD51 [RMV-7]	161Dy	1/100	104102	Biolegend
MCAM [ME-9F1]	162Dy	1/100	134702	Biolegend
SSEA1 [MC-480]	163Dy	1/100	125602	Biolegend
PDGFRB [APB5]	164Dy	1/100	14-1402-82	EBioScience
CD59a [mCD59.3]	165Ho	1/100	143104	Biolegend
CD104 [346-11A]	166Er	1/100	123602	Biolegend
CD54 [YN1/1.7.4]	167Er	1/100	116102	Biolegend
CD47 [miap301]	168Er	1/100	127502	Biolegend
CD90 [G7]	169Tm	1/100	105202	Biolegend

CD73 [TY/11.8]	170Er	1/100	127202	Biolegend
CD133 [315-2C11]	171Yb	1/100	141202	Biolegend
CD14 [M14-23]	172Yb	1/100	150102	Biolegend
CD29 [HMb1-1]	173Yb	1/100	14-0291-85	EBioScience
EpCAM [G8.8]	174Yb	1/100	14-5791-85	EBioScience
CD98 [RL388]	175Lu	1/100	128202	Biolegend
Podoplanin [8.1.1]	176Yb	1/100	127402	Biolegend
I-A/I-E [M5/114.15.2]	209Bi	1/100	3209006B	Fluidigm
CD45 [30-F11]	89Y	1/100	3089005B	Fluidigm
