# Supplementary material for: A transcriptional response to replication stress selectively expands a subset of Brca2-mutant mammary epithelial cells
Source: Nat Commun. 2023 Aug 25;14:5206. doi: 10.1038/s41467-023-40956-w (PMC10457340; doi:10.1038/s41467-023-40956-w)
Supplement: Supplementary file 3 — Description of Additional Supplementary Files [file 41467_2023_40956_MOESM3_ESM.pdf]

**Description of Additional Supplementary Files**

File Name: Supplementary Data 1
Description: Differentially expressed genes between Passage 4 HU-treated Brca2mut/WT cells and wildtype control, HU-treated wildtype and Brca2mut/WT control cells.

File Name: Supplementary Data 2
Description: Gene ontology GO and MSigDB terms for top differentially expressed genes DEGs upregulated in Brca2mut/WT HU treated cellscompared to wildytpe (control and HU treated) and Brca2mut/WT control.

File Name: Supplementary Data 3
Description: Gene ontology GO terms for top differentially expressed genes DEGs upregulated in WT cells.
