# Supplementary material for: A transcriptional response to replication stress selectively expands a subset of Brca2-mutant mammary epithelial cells
Source: Nat Commun. 2023 Aug 25;14:5206. doi: 10.1038/s41467-023-40956-w (PMC10457340; doi:10.1038/s41467-023-40956-w)
Supplement: Supplementary file 7 — Reporting Summary [file 41467_2023_40956_MOESM7_ESM.pdf]

nature portfolio

Corresponding author(s): Mona Shehata

Last updated by author(s): Aug 6, 2023

Reporting Summary

Nature Portfolio wishes to improve the reproducibility of the work that we publish. This form provides structure for consistency and transparency in reporting. For further information on Nature Portfolio policies, see our Editorial Policies and the Editorial Policy Checklist.

Statistics

For all statistical analyses, confirm that the following items are present in the figure legend, table legend, main text, or Methods section.

n/a	Confirmed	
☐	☒	The exact sample size (*n*) for each experimental group/condition, given as a discrete number and unit of measurement
☐	☒	A statement on whether measurements were taken from distinct samples or whether the same sample was measured repeatedly
☐	☒	The statistical test(s) used AND whether they are one- or two-sided<br>*Only common tests should be described solely by name; describe more complex techniques in the Methods section.*
☒	☐	A description of all covariates tested
☐	☒	A description of any assumptions or corrections, such as tests of normality and adjustment for multiple comparisons
☐	☒	A full description of the statistical parameters including central tendency (e.g. means) or other basic estimates (e.g. regression coefficient) AND variation (e.g. standard deviation) or associated estimates of uncertainty (e.g. confidence intervals)
☐	☒	For null hypothesis testing, the test statistic (e.g. *F*, *t*, *r*) with confidence intervals, effect sizes, degrees of freedom and *P* value noted<br>*Give P values as exact values whenever suitable.*
☒	☐	For Bayesian analysis, information on the choice of priors and Markov chain Monte Carlo settings
☒	☐	For hierarchical and complex designs, identification of the appropriate level for tests and full reporting of outcomes
☒	☐	Estimates of effect sizes (e.g. Cohen's *d*, Pearson's *r*), indicating how they were calculated

Our web collection on statistics for biologists contains articles on many of the points above.

Software and code

Policy information about availability of computer code

Data collection

Axio Lab A1 microscope, Canon EOS 1000D camera with AxioVision software (Carl Zeiss), Zeiss 880 confocal with Zeiss Z1.imager – software Zen2 6.1.7601 were used for image collection. Included in methods section.
Operetta CLS inverted high-content imaging system Detection: PerkinElmer Operetta CLS built-in camera (Andor Zyla 5.5). Acquisition software: PerkinElmer Harmony 4.9

FACS Fortessa (BD Biosciences) – BD FACS Diva v8 software
Novaseq 6000 S2 (Illumina) and Cell Ranger pipeline 6.0.2 (10× Genomics) were used for single-cell sequencing data collection.

Data analysis

The single cell RNA sequencing bioinformatics analyses were conducted using open-source software, including Seurat version V4.0, R version 4.1.3. Data code is available from github (https://github.com/ms2140/Brca2_mammary; https://zenodo.org/badge/latestdoi/542765593). Flow cytometry data was analysed using FLowJo_V10 software (FLowJo LLC, Ashland, U.S.). Wholemount immunofluorescent images were analysed with ImageJ version 1.53q. Data representation and analyses was conducted with Graphpad PRISIM 9. Heat maps for CyTOF data were generated in R version 3.5.1. The X-shift algorithm in the Vortex software package was used to analyse CyTOF data.

For manuscripts utilizing custom algorithms or software that are central to the research but not yet described in published literature, software must be made available to editors and reviewers. We strongly encourage code deposition in a community repository (e.g. GitHub). See the Nature Portfolio guidelines for submitting code & software for further information.

Data

Policy information about availability of data

All manuscripts must include a data availability statement. This statement should provide the following information, where applicable:

- Accession codes, unique identifiers, or web links for publicly available datasets
- A description of any restrictions on data availability
- For clinical datasets or third party data, please ensure that the statement adheres to our policy

The single-cell RNA-seq data generated in this study have been deposited in NCBI GEO and can be accessed using the GSE214539 accession number. The CyTOF data generated in this study have been deposited in Mendeley Data (DOI:10.17632/3nm9wnbndc.1). GO terms libraries from Enrichr (https://maayanlab.cloud/Enrichr/#libraries) including GO_Biological_Process_2021; MSigDB_Hallmark_2020 and KEGG_2019_Mouse were used for the analysis. BRCA2 mutation status were collated from cBioPortal (https://www.cbioportal.org/) querying TCGA-BRCA datasets. Hallmark geneset was used from Molecular Signature Database (https://www.gsea-msigdb.org/gsea/msigdb). All other relevant data are provided within the articles and supplementary information files and source data file.

Research involving human participants, their data, or biological material

Policy information about studies with human participants or human data. See also policy information about sex, gender (identity/presentation), and sexual orientation and race, ethnicity and racism.

Reporting on sex and gender	n/a
Reporting on race, ethnicity, or other socially relevant groupings	n/a
Population characteristics	n/a
Recruitment	n/a
Ethics oversight	n/a

Note that full information on the approval of the study protocol must also be provided in the manuscript.

Field-specific reporting

Please select the one below that is the best fit for your research. If you are not sure, read the appropriate sections before making your selection.

☒ Life sciences ☐ Behavioural & social sciences ☐ Ecological, evolutionary & environmental sciences

For a reference copy of the document with all sections, see nature.com/documents/nr-reporting-summary-flat.pdf

Life sciences study design

All studies must disclose on these points even when the disclosure is negative.

Sample size	No statistical methods were used to predetermine sample size. Sample size in the mouse organoid studies were based on preliminary experimentation and previous published mammary organoid results. We designed our experiment to achieve a minimum n=3, but mostly n=5 (or greater) mice per genotype to generate the organoids which would then be used per condition. This enabled us to quantify the data and report biologically significant experiments with reproducible results.
Data exclusions	No data were excluded
Replication	All experiments (organoids, single cell RAN-sequencing, CRISPR etc) were performed using at least three biological independent samples, except if otherwise stated. The exact number of biological replicates for each experiment are indicated in the figure and figure legends.
Randomization	Female wildtype or Brca2mut/WT mice between the ages of 3 months and 8-11 months were randomly selected and mammary glands collected. For all experiments were there were different conditions, organoids generated were randomly attributed to a condition.
Blinding	Mouse IDs were used for naming organoid cultures instead of the genotypes. The experimenter was blinded to the genotype during data collection and analysis. For the different treatment conditions, the experiments were not blinded during data collection or analysis.

Reporting for specific materials, systems and methods

We require information from authors about some types of materials, experimental systems and methods used in many studies. Here, indicate whether each material, system or method listed is relevant to your study. If you are not sure if a list item applies to your research, read the appropriate section before selecting a response.

Materials & experimental systems

n/a	Involved in the study
☐	☒ Antibodies
☒	☐ Eukaryotic cell lines
☒	☐ Palaeontology and archaeology
☐	☒ Animals and other organisms
☒	☐ Clinical data
☒	☐ Dual use research of concern
☒	☐ Plants

Methods

n/a	Involved in the study
☒	☐ ChIP-seq
☐	☒ Flow cytometry
☒	☐ MRI-based neuroimaging

Antibodies

Antibodies used

The following primary, secondary and conjugated antibodies are included in Supplemental Data File and referred in methods sections. Chicken polyclonal anti-Keratin 14 (#906004, BioLegend, 1:1000), Mouse monoclonal anti-Progesterone Receptor (#66300-1-IG, Proteintech, 1:100), Rabbit polyclonal anti-CD36 (#18836-1-AP, Proteintech, 1:250), Mouse monoclonal anti-CD14 (#66253-1-Ig, Proteintech, 1:100), Rabbit polyclonal anti-E-Cadherin (#20874-1-AP, Proteintech, 1:500), Mouse monoclonal anti-E-Cadherin (#610181, BD, 1:500), Rabbit polyclonal anti-Vimentin (#ab92547, Abcam, 1:1000), EpCAM-Brilliant Violet 605 (#118227, BioLegend, 1:400), CD49f-PE/Cy7 (#313622, BioLegend, 1:400), CD49b-APC (#103515, BioLegend, 1:100), Sca1- APC/Fire750 (#108145, BioLegend, 1:1000), CD31-Biotin (#13-0311-81, Invitrogen, 1:500), CD45-Biotin (#47-0451-80, Invitrogen, 1:500), Ter119-Biotin, (#116204, BioLegend, 1:500), Tspan8-PE (#FAB6524P, R&D Systems, 1:100), BrdU-FITC (#364105, BioLegend, 1:100), Phospho-Histone H2A.X (Ser139)-FITC (#9719S, Cell Signalling Technologies, 1:200), TER119-113ln (#116201, Biolegend, 1:100), CD31-115ln (#102502, Biolegend, 1:100), PCAD-141Pr (#AF761, R&D Systems, 1:100), SSEA4-142Nd (#330402, Biolegend, 1:100), Procr-143Nd (#16-2012-83, EBioScience, 1:100), CD200-144Nd (#123802, Biolegend, 1:100), Sca1-145Nd (#122502, Biolegend, 1:100), EGFR146Nd (#54359S, Cell Signaling, 1:100), Kit-147Sm (#105802, Biolegend, 1:100), CD49f-148Nd (#313602, Biolegend, 1:100), CD9-149Sm (#124802, Biolegend, 1:100), CD44-150Nd (#103002, Biolegend, 1:100), CD61-151Eu (#104302, Biolegend, 1:100), ALCAM-152Sm (#14-1661-82, EBioScience, 1:100), CD36-154Sm (#102602, Biolegend, 1:100), CD66a-153Eu (#134506, Biolegend, 1:100), ECAD-155Gd (#AF748, R&D Systems, 1:100), Tspan8-156Gd (#MAB6524, R&D Systems, 1:100), CD24-158Gd (#11-0242-85, EBioScience, 1:100), CD49b-159Tb (#103501, Biolegend, 1:100), Ly6D-160Gd (#557360, BD Bioscience, 1:100), CD51-151Dy (#104102, Biolegend, 1:100), MCAM-162Dy (#134702, Biolegend, 1:100), SSEA1-163Dy (#125602, Biolegend, 1:100), PDGFRB-164Dy (#14-1402-82, EBioScience, 1:100), CD59a-165Ho (#143104, Biolegend, 1:100), CD104-166Er (#123602, Biolegend, 1:100), CD54-167Er (#116102, Biolegend, 1:100), CD47-168Er (#127502, Biolegend, 1:100), CD90-169Tm (#105202, Biolegend, 1:100), CD73-170Er (#127202, Biolegend, 1:100), CD133-171Yb (#141202, Biolegend, 1:100), CD14-172Yb (#150102, Biolegend, 1:100), CD29-173Yb (#14-0291-85, EBioScience, 1:100), EpCAM-174Yb (#14-5791-85, EBioScience, 1:100), CD98-175Lu (#128202, Biolegend, 1:100), Podoplanin-176Yb (#127402, Biolegend, 1:100), I-A/I-E-209Bi (#3209006B, Fluidigm, 1:100), CD4589Y (#3089005B, Fluidigm, 1:100), Goat- anti-Chicken AF488 (#103-545-155, Jackson ImmnunoResearch, 1:500), Goat anti-Mouse AF647 (#115-545-166, Jackson ImmnunoResearch, 1:500), Goat anti-Rabbit Cy3 (#111-165-003, Jackson ImmnunoResearch, 1:500).

Validation

All antibodies for immunofluorescence, flow cytometry and mass cytometry are from commercial sources and vendor confirmed species reactivity.
Chicken polyclonal anti-Keratin 14 (Alex Fluor 488) (#906004, BioLegend) was validated by confirming tissue staining in basal mammary epithelial cells in tissue samples, but absence of staining in appropriate luminal and stromal cells by immunofluorescence. Mouse monoclonal anti-Progesterone Receptor (Alexa Fluor 647) (#66300-1-IG, Proteintech) is noted on the manufacturer's website to be validated for assays including immunohistochemistry and immunofluorescence. Validated by confirming nuclear immunostaining in luminal mammary epithelial cells. Rabbit polyclonal anti-CD36 (Cy3) (#18836-1-AP, Proteintech) is noted on the manufacturer's website to be validated for assays including immunohistochemistry. Validated by confirming cell membrane immunostaining in mammary epithelial cells. Mouse monoclonal anti-CD14 (Alexa Fluor 647) (#66253-1-Ig, Proteintech) is noted on the manufacturer's website to be validated for assays including immunohistochemistry and immunofluorescence and tested in mouse. Validated by confirming cell membrane immunostaining in basal mammary epithelial cells. Rabbit polyclonal anti-E-Cadherin (Cy3) (#20874-1-AP, Proteintech) is noted on the manufacturer's website to be validated for assays including immunohistochemistry and immunofluorescence and tested in mouse. Validated by confirming cell membrane immunostaining in mammary epithelial cells. Mouse monoclonal anti-E-Cadherin (Alexa Fluor 647) (#610181, BD) is noted on the manufacturer's website to be validated for assays including immunohistochemistry and immunofluorescence and tested in mouse. Validated by confirming cell membrane immunostaining in mammary epithelial cells. Rabbit polyclonal anti-Vimentin (Cy3) (#ab92547, Abcam) is noted on the manufacturer's website to be validated for assays including immunohistochemistry and immunofluorescence and tested in mouse. Validated by confirming cell membrane immunostaining in basal mammary epithelial cells. EpCAM-Brilliant Violet 605 (#118227, BioLegend) is noted on the manufacturer's website to be quality tested for flow cytometry. This antibody was further validated for flow cytometry by inspection of biaxial plots for expected staining patterns of mammary epithelial cells. CD49f-PE/Cy7 (#313622, BioLegend) is noted to be quality tested for flow cytometry on the manufacturer's website. This antibody was validated for flow cytometry by inspection of biaxial plots for expected staining patterns of mammary epithelial cells. CD49b-APC (#103515, BioLegend) is noted on the manufacturer's website to be verified and quality tested for flow cytometry in mouse cells. This antibody was validated for flow cytometry by inspection of biaxial plots for expected staining patterns of luminal mammary epithelial cells. Sca1-APC/Fire750 (#108145, BioLegend) is noted on the manufacturer's website to be verified and quality tested for flow cytometry in mouse cells. This antibody was validated for flow cytometry by inspection of biaxial plots for expected staining patterns of luminal mammary epithelial cells. CD31-Biotin (#13-0311-81, Invitrogen) is noted on the manufacturer's website to be verified and quality tested for flow cytometry in mouse cells. This antibody was validated for flow cytometry by inspection of biaxial plots for expected staining patterns of mammary haemopoietic/endothelial cells. CD45-Biotin (#47-0451-80, Invitrogen) is noted on the manufacturer's website to be verified and quality tested for flow cytometry in mouse cells. This antibody was validated for flow cytometry by inspection of biaxial plots for expected staining patterns of mammary haemopoietic/endothelial cells. Ter119-Biotin, (#116204, BioLegend) is noted on the manufacturer's website to be verified and quality tested for flow cytometry in mouse cells. This antibody was validated for flow cytometry by inspection of biaxial plots for expected staining patterns of mammary haemopoietic/endothelial

cells. Tspan8-PE (#FAB6524P, R&D Systems) is noted on the manufacturer's website to be verified and quality tested for flow cytometry in mouse cells. This antibody was validated for flow cytometry by inspection of biaxial plots for expected staining patterns of mammary basal and luminal epithelial cells. BrdU-FITC (#364105, BioLegend) is noted on the manufacturer's website to be verified and quality tested for intracellular flow cytometry. This antibody was validated for intracellular flow cytometry by inspection of biaxial plots on mammary epithelial cells. Phospho-Histone H2A.X (Ser139)-FITC (#9719S, Cell Signalling Technologies) is noted on the manufacturer's website to be verified and quality tested for intracellular flow cytometry. This antibody was validated for intracellular flow cytometry by inspection of biaxial plots on mammary epithelial cells. For all CyTOF experiments, all the following antibodies was validated by performing flow cytometry on mouse mammary cells and by performing mass cytometry on mammary cells by inspection of boaxial plots :TER119 (#116201, Biolegend), CD31 (#102502, Biolegend), PCAD (#AF761, R&D Systems), SSEA4 (#330402, Biolegend), Procr (#16-2012-83, EBioScience), CD200 (#123802, Biolegend), Sca1 (#122502, Biolegend), EGFR (#54359S, Cell Signaling), Kit (#105802, Biolegend), CD49f (#313602, Biolegend), CD9 (#124802, Biolegend), CD44 (#103002, Biolegend), CD61 (#104302, Biolegend), ALCAM (#14-1661-82, EBioScience), CD36 (#102602, Biolegend), CD66a (#134506, Biolegend), ECAD (#AF748, R&D Systems), Tspan8 (#MAB6524, R&D Systems), CD24 (#11-0242-85, EBioScience), CD49b (#103501, Biolegend), Ly6D (#557360, BD Bioscience), CD51 (#104102, Biolegend), MCAM (#134702, Biolegend), SSEA1 (#125602, Biolegend), PDGFRB (#14-1402-82, EBioScience), CD59a (#143104, Biolegend), CD104 (#123602, Biolegend), CD54 (#116102, Biolegend), CD47 (#127502, Biolegend), CD90 (#105202, Biolegend), CD73 (#127202, Biolegend), CD133 (#141202, Biolegend), CD14 (#150102, Biolegend), CD29 (#14-0291-85, EBioScience), EpCAM (#14-5791-85, EBioScience), CD98 (#128202, Biolegend), Podoplanin (#127402, Biolegend), I-A/I-E (#3209006B, Fluidigm) and on the manufacturer's website is noted to be quality control tested by CyTOF analysis, CD45 (#3089005B, Fluidigm) and on the manufacturer's website is noted to be quality control tested by CyTOF analysis.

Animals and other research organisms

Policy information about studies involving animals; ARRIVE guidelines recommended for reporting animal research, and Sex and Gender in Research

Laboratory animals	Laboratory animals: Mus musculus. Brca2 Tr/WT19 and littermate Brca2 WT/WT strains between 3-10 months old. Female mice were used for this study. Animals were housed in HMS animal facility, in temperatures between 20-24°C, 40-70% humidity and a 12h light/dark cycle.
Wild animals	This study did not involve wild animals
Reporting on sex	This study investigates a gender specific organ, the mammary gland. As such this study only uses female mice.
Field-collected samples	This study did not involve samples collected from the field.
Ethics oversight	All mice were treated in strict accordance with the local ethical committee (University of Cambridge License Review Committee) and the UK Home Office guidelines.

Note that full information on the approval of the study protocol must also be provided in the manuscript.

Flow Cytometry

Plots

Confirm that:

☒ The axis labels state the marker and fluorochrome used (e.g. CD4-FITC).

☒ The axis scales are clearly visible. Include numbers along axes only for bottom left plot of group (a 'group' is an analysis of identical markers).

☒ All plots are contour plots with outliers or pseudocolor plots.

☒ A numerical value for number of cells or percentage (with statistics) is provided.

Methodology

Sample preparation	Mammary epithelial cells were collected from third and fourth mammary glands of virgin mice. Briefly, lymph nodes were removed, and glands minced with surgical scissors before enzymatical dissociation for 1.5h in DMEM/F12 (1:1) supplemented with 2 mg mL−1 collagenase (Roche) + Gentamicin (Gibco). Samples were briefly vortexed every 30min. Mammary gland fragments were treated with NH4CL to lyse red blood cells, then dissected to single cells with 0.05% Trypsin-EDTA (STEMCELL Technologies) and 5 mg ml-1 dispase (STEMCELL Technologies) and 1 mg ml-1 DNAse (Sigma) and filtered through a 40 µm cell strainer (Falcon).<br>Mammary organoids were mechanical dissociated in TrypLE for 10-15 minutes at 37°C to generate a single cell suspension.
Instrument	FACS Fortessa (BD Biosciences)
Software	BD FACS Diva v8 software. Data was analysed using FLowJo_V10 software (FLowJo LLC, Ashland, U.S.).
Cell population abundance	Abundance (percentage) is indicated for the relevant populations on flow cytometry plots.
Gating strategy	For primary mammary glands: Cells were first identified using FSC/SSC, then singlets were identified using FSC-W/FSC-A and then SSA-W/SSA-A . Live cells were identified using FSC/DAPI and endothelial cells were excluded using FSC/Lin (Ter119,

CD31, CD45). Luminal and Basal mammary epithelial cells were identified using EPCAM and CD49f. The luminal population was further fractionated into subpopulations using Sca1 and CD49b. This gating strategy has been utilised previously (Shehata et al. Breast Cancer Res 14, R134, 2012).
For organoids: Cells were first identified using FSC/SSC, then singlets were identified using SSA-W/SSA-A and then FSC-W/FSC-A. Live cells were identified using DAPI/FSC or Zombie-UV/FSC. Luminal and Basal mammary epithelial cells were identified using EPCAM and CD49f. The luminal population was further fractionated into subpopulations using Sca1 and CD49b, and Tspan8. Proliferating cells in the different subpopulations were identified using BrdU and DNA damged cells was identified using Phospho-Histone H2A.X.

☒ Tick this box to confirm that a figure exemplifying the gating strategy is provided in the Supplementary Information.
